# A New Ursane-Type Pentacyclic Triterpenoid from the Tree Bark of *Sandoricum koetjape*: Antibacterial, DFT, and Molecular Docking Study

**DOI:** 10.3390/ijms262110389

**Published:** 2025-10-25

**Authors:** Husnul Khatimah, Elvira Hermawati, Fadjar Mulya, Muhammad Ikhlas Abdjan, Thanawit Kuamit, Ade Danova

**Affiliations:** 1 Organic Chemistry Division, Department of Chemistry, Faculty of Mathematics and Natural Sciences, Institut Teknologi Bandung, Jl. Ganesha No.10, Bandung 40132, Indonesia; husnulkhatimah52@gmail.com; 2 Nanotechnology Engineering Program, Faculty of Advanced Technology and Multidiscipline, Universitas Airlangga, Surabaya 60115, Indonesia; fadjar.mulya@ftmm.unair.ac.id; 3 Department of Chemistry, Faculty of Mathematics and Natural Sciences, Universitas Negeri Surabaya, Surabaya 60213, Indonesia; muhammadabdjan@unesa.ac.id; 4 Center of Excellence in Computational Chemistry (CECC), Department of Chemistry, Faculty of Science, Chulalongkorn University, Bangkok 10330, Thailand; thanawit.kuamit@chula.ac.th

**Keywords:** antibacterial, density functional calculation, docking study, *Sandoricum koetjape*, terpenoids

## Abstract

Natural products have played an important role in the discovery and development of antibacterial agents. This paper described the isolation of a new ursane-type pentacyclic triterpenoid, (18β,19αH)-3-oxo-urs-12-en-27α-oic acid (**2**), from the tree bark of *Sandoricum koetjape* Merr. Along with this, five known compounds—β-caryophyllene oxide (**1**), bryononic acid (**3**), 7-deacetylgedunin (**4**), 7-deacetyl-7-oxogedunin (**5**), and 12,20-dihydroxydammar-24-en-3-one (**6**)—were successfully isolated, and one compound, 12β-hydroxydammarenolic acid (**7**), was reported in our previous report. All compounds (**1**–**7**) were tested with their antibacterial properties against two Gram-positive (*Enterococcus faecalis* and *Staphylococcus saprophyticus*) and two Gram-negative (*Citrobacter freundii* and *Salmonella enterica*) bacteria. The structures of the isolated compounds were elucidated using NMR spectroscopy and mass spectrometry data. A preliminary antibacterial assay showed that only compound **7** inhibited the growth of the tested bacteria, with an inhibition zone diameter of 7.5–9 mm at a concentration of 1 mg/mL. DFT analyses explained electronic profiles with HOMO-LUMO gaps (4.54–6.34 eV) and electrophilicity from 1.73 to 4.39 eV. To elucidate the antibacterial mechanism of compound **7**, a molecular docking study was conducted. The findings from both in vitro and in silico analyses suggest that compound **7** is a promising antibacterial candidate for further investigation.

## 1. Introduction

Since the discovery of penicillin in 1928, antibiotics have been crucial for public health, saving the lives of millions of people worldwide. However, in recent years, it has been discovered that an increasing number of bacteria are developing resistance [1]. Antibacterial resistance is a global public health challenge that is thought to have contributed to 4.95 million deaths in 2019, primarily in low- and middle-income countries [2]. Antibiotic resistance has been reported in bacteria that cause common diseases such as urinary tract infections, pneumonia, bloodstream infections, skin infections, etc. [3]. In 2024, the World Health Organization (WHO) published a list of antibiotic-resistant priority pathogens, which pose a significant threat to people, and therefore new treatments are urgently needed. Gram-negative bacteria that are resistant to last-resort antibiotics, like *Acinetobacter baumannii* and various *Enterobacter pathogens*, as well as *Mycobacterium tuberculosis*, are classified as critical priority due to their ability to transfer resistance genes. Other bacteria, including *Salmonella*, *Shigella*, *Pseudomonas aeruginosa*, *Staphylococcus aureus*, *Neisseria gonorrhoeae*, and *Enterococcus faecium,* are categorized as a high priority because of their resistance to existing treatments and the significant burden of infection associated with these pathogens [2].

Natural products have played a crucial role in the discovery and development of antibacterial agents as they continue to provide new chemical structures [4]. For almost four decades, from 1981 to 2019, around 48% of approved antibacterial agents were derived from natural products and derivatives [5]. Terpenes and their derivatives have been studied and found to have significant antimicrobial activities, demonstrating bacteriostatic and bactericidal effects against tested pathogens through various mechanisms of action [6]. Pentacyclic triterpenoids, including oleanolic and ursolic acids, isolated from *Alstonia scholaris* showed significant antibacterial activity against Gram-positive bacteria, *E. faecalis*, *L. monocytogenes*, and *B. cereus* [7]. Recently, a series of semisynthetic triterpenoids with A-ring azepano- and A-seco-fragment substituents at C3 and C28 demonstrated a strong bacteriostatic effect against Methicillin-resistant *S. aureus* (MIC  ≤  0.15 μM), exceeding the clinically used antibiotic vancomycin (MIC  =  0.625 μM) [8]. Moreover, an oleanane derivative also showed significant antimicrobial activity against both Gram-positive and Gram-negative bacteria [9].

*Sandoricum koetjape* Merr., a species of Meliaceae, has some potential secondary metabolites. Traditionally, plants have been used to address digestive issues and inflammatory diseases [10]. Phytochemical studies of the leaves, seeds, fruit hulls, stem barks, and barks of *S. koetjape* have revealed the presence of terpenoids, predominantly pentacyclic triterpenoids and limonoids [11,12,13,14,15,16,17,18,19,20,21,22]. Pentacyclic triterpenoids were dominated by oleanane- and multiflorane-type triterpenes, while the limonoids were dominated by andirobin and trijugin types. Some of these compounds have been shown to be effective antifeedants [20] and anti-inflammatory agents [18], and to exhibit potential cytotoxicity against several cancer cells [11,12,14,21]. Recently, a tirucallane-type tetracyclic triterpene isolated from the seed and fruit peels of *S. koetjape* exhibited weak activity against InsR [13]. Additionally, two new cycloartane-type tetracyclic triterpenes isolated from the leaves also showed promising α-glucosidase inhibitory activity [23]. To date, the isolation of secondary metabolites from the tree bark of *S. koetjape* remains limited. Previously, we reported the isolation of a new seco-dammarane triterpene, 12β-hydroxydammarenolic acid (**7**), and its inhibitory properties against several receptor tyrosine kinases [24]. In searching for antibacterial agents from natural products, in this study, we report our isolation, structural elucidation, and preliminary biological evaluation against four bacteria of a new pentacyclic triterpenoid (**2**) and five known compounds: limonoids (**4**, **5**), triterpenoids (**3**, **6**), and sesquiterpenoid (**1**) from the tree bark of MeOH extract of *S. koetjape* from Pandeglang, Banten, Indonesia. Furthermore, Density Functional Theory (DFT) calculations were performed to study the reactivity and stability of all compounds (**1**–**7**), and molecular docking analysis was conducted to study the molecular interaction between a potent compound (**7**) and PTP1B as the targeted protein.

## 2. Results

### 2.1. Identification of Isolated Compounds from S. koetjape

**Figure 1:**
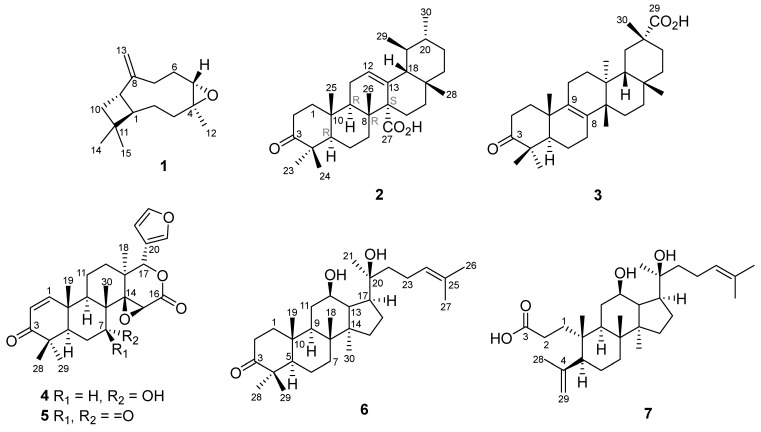
Structures of isolated compounds **1**–**7**.

The tree bark powders of *S. koetjape* were dried and extracted with MeOH, then fractionated and purified using chromatographic techniques to obtain β-caryophyllene oxide (**1**), (18β,19αH)-3-oxo-urs-12-en-27α-oic acid (**2**), bryononic acid (**3**), 7-deacetylgedunin (**4**), 7-deacetyl-7-oxogedunin (**5**), 12,20-dihydroxydammar-24-en-3-one (**6**), and 12β-hydroxydammarenolic acid (**7**) (Figure 1), which have been reported against eight receptor tyrosine kinases [24]. Two gedunin-type limonoids (**4**, **5**) were first isolated from *S. koetjape*, although these compounds have been isolated from other Meliaceae species [25,26]. The dammarane-type triterpenes (**6**, **7**) [24] were also isolated from this plant for the first time, whereas tetracyclic triterpenoids have rarely been found in this plant. Moreover, this is the first report of the occurrence of **2** as a new ursane-type pentacyclic triterpene.

**Figure 2:**
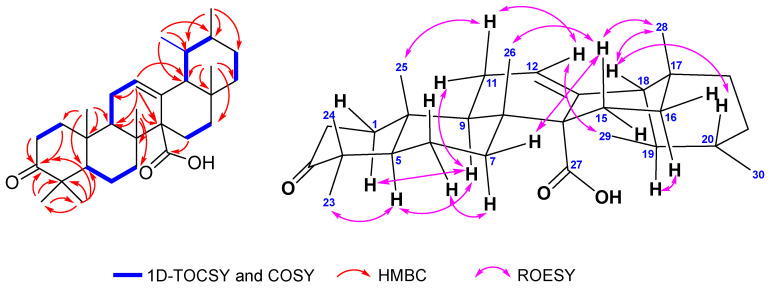
TOCSY-1D, HMBC, and ROESY correlation of **2**.

Compound **2** was obtained as an optically active ([α]^23^_D_ +142.0, c 0.1; CHCl_3_) white powder. HRESIMS analysis revealed its molecular formula as C_30_H_46_O_3_, with a molecular ion peak [M-H]^−^ at *m*/*z* 453.3367 (calcd. 453.3369). The ^13^C-NMR spectrum (Table 1; Figure S8) showed 30 carbon signals, including a trisubstituted alkene (δ_C_ 128.4 and 133.2), a carboxylic group (δ_C_ 180.9), a ketonic carbonyl (δ_C_ 217.9), a pair of vicinal methyls (δ_C_ 17.8 and 21.4), five quaternary sp^3^ carbons (δ_C_ 47.1; 39.8; 36.6; 55.8; 33.7), and five additional methyl groups (δ_C_ 27.0; 21.3; 16.4; 18.1; 29.0). These functionalities represent three of the eight degrees of unsaturation, suggesting that **2** has a pentacyclic skeleton. The ^1^H-NMR spectrum (Table 2; Figures S6 and S7) exhibited five singlet methyl signals (δ_H_ 1.06; 1.03; 1.05; 1.07; 0.84), one doublet methyl (δ_H_ 0.79), a broad singlet methyl (δ_H_ 0.86) in the upfield region, and an olefinic proton (δ_H_ 5.58) in the downfield region, which are typical for ursane-type triterpene [27,28]. The ketonic carbonyl was assigned to C-3 based on the HMBC correlations of H-1, H-2, and the pair of gem-dimethyl protons (H-23 and H-24) with the carbonyl group. The HMBC correlation between δ_H_ 1.77 (H-15β), δ_H_ 1.98 (H-15α), and δ_C_ 180.9 (C-27) indicated that the carboxylic acid group was attached to C-14. Furthermore, the absence of methyl proton correlation with the olefinic carbon also supported that C-27 was oxidized to the carboxylic acid group (Figure 2). The relative stereochemistry was further elucidated based on proton coupling constants and NOE correlations observed in the ROESY spectrum (Figure S11, Table S1). The α-axial orientation of H-9 was deduced from its coupling constant (*J* = 11.0 and 5.45 Hz). The larger *J* value corresponds to trans-diaxial coupling, whereas the smaller one indicates an axial-equatorial interaction with H-11, thereby confirming the axial orientation of H-9. The cross-peaks between H-5/H-9, H-5/H-23, and H-9/H-11α indicated that they were on the same face in the α-configuration. In addition, the axial orientation of H-15β (δ_H_ 1.77) was inferred from the contour pattern. NOE correlations between H-15β/H-26, H-15β/H-28, H-28/H-18, and H-18/H-20 suggested that they were on the β-configuration. Furthermore, the methine proton at δ_H_ 2.11 (td, *J* = 13.62; 3.85 Hz, H-16) indicated an α-axial orientation. This proton showed a NOESY correlation with H-19, suggesting that H-19 is also in the α-configuration, whereas C-29 is β-oriented. This assignment was further supported by the observed NOESY correlation between H-29 and H-12 olefinic proton. Detailed analysis of 1D and 2D NMR spectra indicated that compound **2** possessed a skeleton similar to that of (14*S*)-3-oxo-18α,19αH-urs-12-en-27α-oic acid [29], differing in the β-axial orientation of H-18 (Figure 2). The absolute configurations at C-5, C-8, C-9, and C-14 were determined to be 5*R*, 8*R*, 9*R*, and 14*S*, respectively, based on a comparison with previously reported ursane-type triterpenoids [30], which is consistent with the stereochemistry of the α-amyrin-derived ursane skeleton. Thus, compound **2** was assigned as (18β,19αH)-3-oxo-urs-12-en-27α-oic acid. To the best of our knowledge, this is the first report of an ursane-type pentacyclic triterpenoid isolated from *S. koetjape*. Moreover, the oxygenation at C-27 is also rarely found in this plant.

### 2.2. Antibacterial Evaluation

Preliminary antibacterial evaluation of all isolated compounds, prepared at a concentration of 1 mg/mL (equivalent to 10 μg per disk), against two Gram-positive (*E. faecalis* and *S. saprophyticus*) and two Gram-negative (*C. freundii* and *S. enterica*) bacteria showed that only **7** inhibited the growth of the tested bacteria (Table 3).

### 2.3. Study of Density Functional Theory (DFT)

To further explore these reactivity parameters, Density Functional Theory (DFT) calculations on the B3LYP/6-31+g(d,p) level were conducted on compounds **1**–**7**. These results, summarized in Table 4, provide insight into important reactivity descriptors like the HOMO-LUMO energy gap, chemical hardness, and electrophilicity index. The HOMO-LUMO energy gap is a vital factor in determining the reactivity and stability of a molecule. A smaller gap signifies higher reactivity and lower stability, while a larger gap suggests greater stability and reduced reactivity.

Our results indicate that compound **5** has the smallest HOMO-LUMO energy gap (4.54 eV), making it the most reactive and least stable molecule in the set. In contrast, Compound **1**, with the largest HOMO-LUMO energy gap (6.34 eV), is the most stable but exhibited lower reactivity. This large gap in Compound **1** limits its ability to undergo electronic transitions, which decreases its reactivity.

Other compounds, such as **2**, **3**, **6**, and **7**, exhibit intermediate values for the HOMO-LUMO gap, ranging from 5.59 to 5.87 eV, indicating moderate reactivity. The electrophilicity indices for these compounds fall between 1.96 and 2.29 eV, suggesting they have a moderate tendency to accept electrons. Their ionization potential and electron affinities also support this, ranging from 6.17 to 6.56 eV and 0.46 to 0.76 eV, respectively. These compounds are, therefore, characterized by a balance of stability and reactivity, making them versatile candidates for various chemical applications.

Compound **5** is characterized by high reactivity and low stability, as evidenced by its small HOMO-LUMO energy gap and high electrophilicity index (4.39 eV), along with a significant ionization potential (6.74 eV) and electron affinity (2.20 eV). Meanwhile, compound **1**, with a low electrophilicity index (1.73 eV), low ionization potential (6.49 eV), and low electron affinity (0.14 eV), is the most stable and least reactive compound.

### 2.4. Molecular Docking Study

PTP1B plays a role in microbial signaling pathways, including cell growth, migration, communication, and gene transcription [31,32]. Owing to these regulations, PTP1B protein is a reliable target protein for understanding the molecular inhibitory mechanisms of small-molecule compounds as antibacterial agents [32]. Moreover, compound **7** was selected because of its promising activity against *E. faecalis* compared to the isolated compounds (**1**–**6**) and chloramphenicol as a control (Table 3). Therefore, compounds **7** and chloramphenicol were modeled to investigate their interactions at the molecular level.

**Figure 3:**
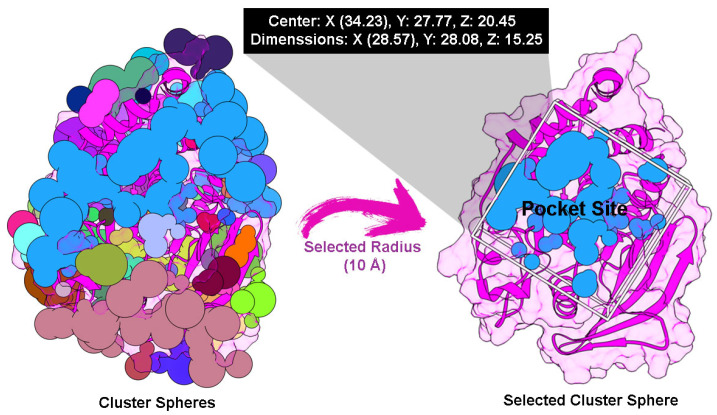
Grid-box preparation: The cluster spheres represent the possibility of a binding site. The selected cluster sphere represents the PTP1B pocket site (blue). The box parameters, such as the center and dimensions, were determined by coordinating the selected spheres.

**Figure 4:**
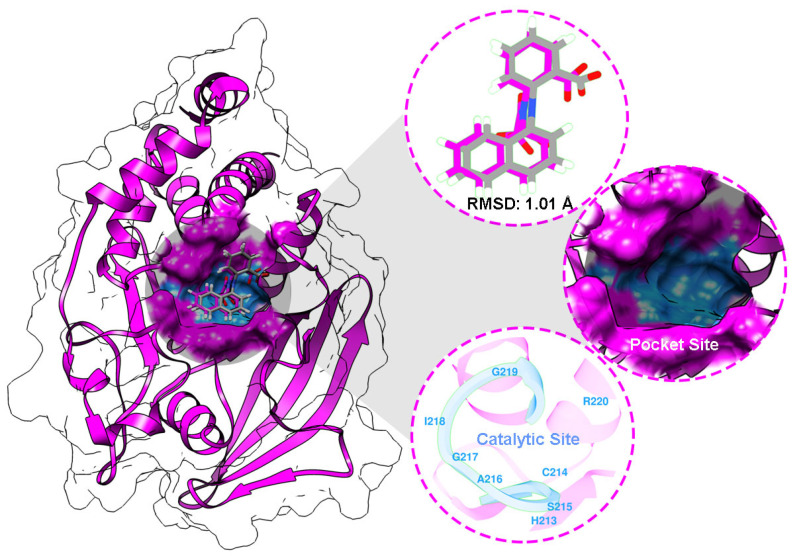
Redocking step: Protein Tyrosine Phosphatase 1B (PTP1B) crystal structure as a targeted protein. Superposition of the native ligand between the crystal (purple) and pose (grey). PTP1B pocket and catalytic sites (blue).

Cluster spheres were built along the targeted protein surface to determine the possibility of pocket site coordination. The accurate coordinates were then selected based on the native ligand coordinates as a reference with a radius of 10 Å. Through the coordination, the grid box was prepared to obtain the PTP1B pocket site (Figure 3). The superposition shows a root-mean-square deviation (RMSD) of 1.01 Å (Figure 4). This finding indicates that the parameters utilized in the redocking step can be used as coordinates on the PTP1B pocket site. This validation also confirmed several amino acids that act as catalytic sites (CS), namely H213, C214, S215, A216, G217, I218, G219, and R220 [32]. As expected, the interaction between the modeled small molecules and the CS region can provide information about the inhibitory mechanism of the active compound’s antibacterial activity.

**Figure 5:**
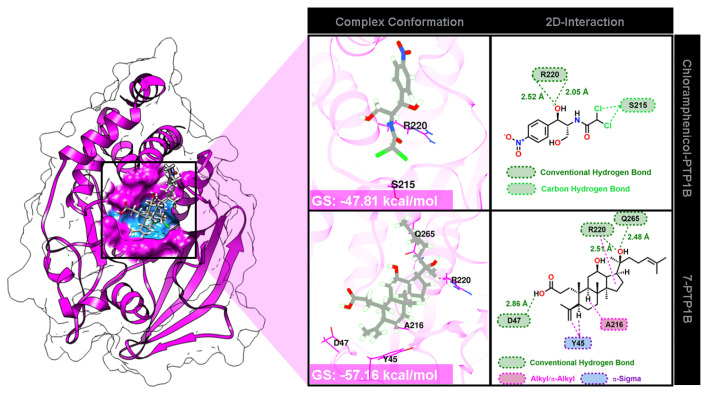
The ligand-PTP1B interaction was presented by complex conformation and 2D interaction.

Docking results revealed that the modeled molecules were well-occupied on the PTP1B binding site (Figure 5). The binding conformation shows that the grid score (GS) forms a stable interaction for chloramphenicol and the compound **7** complex, with the scores of −47.81 and −57.16 kcal/mol, respectively.

## 3. Discussion

In this study, we report the isolation of terpenoids from *S. koetjape*. Among the isolated compounds, only **7** exhibited antibacterial activity. The inhibition zone of **7** against the Gram-positive bacterium *E. faecalis* was slightly larger than that of the positive control (chloramphenicol). Compounds **6** and **7** possess similar skeletons, except for the ring A opening and the existence of a carboxylic acid group in **7**. However, **6** showed no antibacterial activity, suggesting that the ring A seco-triterpenoid with a free carboxylic group found in **7** might be an important factor for antibacterial activity. In a previous study, olenane-type and seco-olenanane pentacyclic triterpenes were tested for antibacterial activities. Among the tested compounds, a seco-oleanane derivative, Koetjapic acid, exhibited the most potent antibacterial activity against *S. aureus* and *P. aeruginosa* [33]. Furthermore, 3,4-seco triterpenoids with a free carboxylic (C-3) isolated from *Dysoxylum hainanense* also showed significant antibacterial activities against some Gram-positive bacteria [34].

The DFT analysis provided valuable information regarding the electronic profiles and reactivities of all compounds. This analysis highlights the potential for tuning the reactivity of molecules by adjusting key electronic properties, such as the HOMO-LUMO energy gap and electrophilicity index, allowing for the design of molecules with specific reactivity profiles for diverse chemical applications.

Docking studies suggested that the favorable binding affinity of compound **7** was stronger than that of chloramphenicol as a control. This finding is related to the in vitro test results shown in Table 3. As mentioned previously, the van der Waals (E_vdW_) and electrostatic (E_ele_) energies contribute to the GS value [35]. The energy contributions of each complex were as follows: chloramphenicol-PTP1B (E_vdW_: −46.56 kcal/mol and E_ele_: −1.25 kcal/mol) and **7**-PTP1B (E_vdW_: −55.70 kcal/mol and E_ele_: −1.45 kcal/mol). As described, E_ele_ contributes significantly to the GS value. Moreover, several interactions with amino acids were confirmed through the PTP1B pocket site (Figure 5). Several molecular interactions were included in each complex, such as conventional hydrogen bonds, alkyl/π-alkyl, π-sigma, and carbon–hydrogen bonds. Interestingly, compound **7** interacted with CS amino acids, including R220 (hydrogen bond) and A216 (π-alkyl). It is known that interaction with amino acid residues around the CS region can block PTP1B enzymatic regulation of microbial growth.

## 4. Materials and Methods

### 4.1. General

Optical rotations were determined using an Autopol IV polarimeter (Rudolph Research Analytical, Hackettstown, NJ, USA). ^1^H-NMR and ^13^C-NMR were recorded with a spectrometer of the Agilent DD2 system (Agilent Technologies, Santa Clara, CA, USA) operating at 500 and 125 MHz, respectively. Mass spectra were measured with Waters LCT Premier XE ESI-TOF-MS (Waters Corporation, Milford, MA, USA). Silica gels (Merck, Darmstadt, Germany) were used for all chromatographic separations. Vacuum Liquid Chromatography (VLC), Centrifugal Planar Chromatography (CPC), and Column Chromatography (CC) were carried out on Merck silica gel G60 254 art. 7731, 7749, and 7733, respectively. Thin-layer chromatography (TLC) analysis was performed using precoated silica gel 60 GF254 plates (0.25 mm thickness; Merck, Darmstadt, Germany). Spots on TLC were detected by UV irradiation and sprayed with CeSO_4_ solution followed by heating. Solvents used for extraction, fractionation, and purification (MeOH, EtOAc, dichloromethane, and *n*-hexane) were of technical grades, which were distilled before use, while CHCl_3_ and diisopropyl ether were of a pro analysis grade. *Enterococcus faecalis* (ATCC 29212), *Staphylococcus saprophyticus* (ATCC 19701), *Citrobacter freundii* (ATCC 13316), and *Salmonella enterica* (ATCC 14028) were obtained from the Microbiology Laboratory, Faculty of Medicine, Universitas Jenderal Soedirman, Purwokerto, Indonesia.

### 4.2. Plant Materials

The tree barks of *S. koetjape* were collected from Pandeglang, Banten Province, Indonesia, in August 2013. Plant identification was performed at the Herbarium Bandungense, Institut Teknologi Bandung (voucher specimen number 11254).

### 4.3. Extraction and Isolation

The dried tree barks of *S. koetjape* powders (1.0 kg) were macerated with MeOH (2 × 10 L, overnight) at room temperature to obtain 61.0 g of dried MeOH extract. Half of the extract (30.0 g) was fractionated into 17 fractions (A–Q) by VLC (silica gel, gradient eluent: *n*-hexane—EtOAc 15% to 70%, then EtOAc and MeOH). Fractions D–E (2.7 g) were separated by VLC eluted with *n*-hexane—EtOAc 15% to 20%, then EtOAc and MeOH generated 14 fractions (DE1–DE14). Fraction DE2 (503.8 mg) was further purified by CPC (eluent: *n*-hexane—CHCl_3_ 10% to 100%) to yield **1** (12.0 mg). Fraction DE14, which was found to be a white solid and showed a single spot on TLC, was filtered to obtain **3** (15.3 mg). Fraction F (1.46 g) was fractionated using VLC (silica gel, gradient eluent: *n*-hexane—EtOAc 5% to 20%, then EtOAc and MeOH) to give 14 fractions (F1–F14). Fractions F11–12 (190.0 mg) were separated by CPC (eluent: *n*-hexane—EtOAc 5% to 20%) then successfully purified using the same method (eluent: *n*-hexane—diisopropyl ether 40%), which afforded **2** (18.5 mg). Fraction L (884.6 mg) was fractionated by CPC using gradient eluent *n*-hexane—EtOAc 20% to 50%, yielding 34 fractions. Fractions L4–L9 (191.9 mg) were separated using a similar method (eluent: *n*-hexane–dichloromethane–EtOAc 7:2:1) afforded **6** (8.5 mg). The fraction then further purified by CC (eluent: CHCl_3_ 100%) gave **4** (8.5 mg). A white solid appeared on fraction N (1.32 g), which showed a minor spot on TLC. The solid then filtered and washed with *n*-hexane to give **5** (27.5 mg). In our previous work, we reported the isolation of **7** from the polar fraction P (1.87 g). A solid (390.0 mg) appeared from the fraction, which showed a minor impurity on the TLC analysis, and which was purified by VLC (eluent: *n*-hexane—EtOAc 20%) to afford **7** (60.0 mg) [24].

β-caryophyllene oxide (**1**)—yellowish oil, [α]^25^_D_ −40.0 (c 0.1; CHCl_3_) ^1^H-NMR (CDCl_3_, 500 MHz), δ_H_ (ppm) 1.76 (1H, t; 9.92 Hz, H-1); 1.43 (1H, m, H-2); 1.65 (1H, m, H-2); 0.97 (1H, m, H-3); 2.09 (1H, dt; 13.15 Hz; 4.19 Hz, H-3); 2.88 (1H, dd; 10.6 Hz; 4.3 Hz, H-5); 2.34 (1H, ddd, 12.70 Hz; 8.07 Hz; 4.46 Hz, H-6); 1.25 (1H, d; 3.58 Hz, H-6); 1.33 (2H, m, H-7); 2.61 (1H, q; 9.5 Hz, H-9); 1.65 (2H, m, H-10); 1.20 (3H, s, H-12); 4.86 (1H, s, H-13); 4.97 (1H, s, H-13); 0.98 (3H, s, H-14); 1.01 (3H, s, H-15). ^13^C-NMR (CDCl_3_, 125 MHz), δ_C_ (ppm) 50.8 (C-1); 27.2 (C-2); 39.2 (C-3); 59.8 (C-4); 63.8 (C-5); 29.8 (C-6); 30.2 (C-7); 151.8 (C-8); 48.7 (C-9); 39.8 (C-10); 34.0 (C-11); 17.0 (C-12); 112.8 (C-13); 29.9 (C-14); 21.6 (C-15). HRESITOF-MS: [M+H]^+^ ion *m*/*z* 221.1908 (calcd. [M+H]^+^ ion for C_15_H_24_O: *m*/*z* 221.1905; Δ 1.4 ppm). The NMR and mass spectra of compound **1** are available in the supplementary file (Figures S1–S5).
(18β,19αH)-3-oxo-urs-12-en-27α-oic acid (**2**)—white powders, [α]^23^_D_ +142.0 (c 0.1; CHCl_3_). ^13^C-NMR (in CDCl_3_) see Table 2; ^1^H-NMR see Table 1. HRESITOF-MS: [M-H]^−^ ion *m*/*z* 453.3367 (calcd. [M-H]^−^ ion for C_30_H_46_O_3_: *m*/*z* 453.3369; Δ 0.4 ppm). The NMR and mass spectra of compound **2** are available in the supplementary file (Figures S6–S12).
Bryononic acid (**3**)—white powders, [α]^23^_D_ +36.0 (c 0.1; CHCl_3_). ^13^C-NMR (in CDCl_3_) see Table 2; ^1^H-NMR see Table 1. HRESITOF-MS: [M-H]^−^ ion *m*/*z* 453.3356 (calcd. [M-H]^−^ ion for C_30_H_46_O_3_: *m*/*z* 453.3369; Δ 2.9 ppm). The NMR and mass spectra of compound **3** are available in the supplementary file (Figures S13–S18).
7-deacetyl gedunin (**4**)—white powders, [α]^23^_D_ +46.0 (c 0.1; CHCl_3_). ^13^C-NMR (in CDCl_3_) see Table 2; ^1^H-NMR see Table 1. HRESITOF-MS: [M+H]^+^ ion *m*/*z* 441.2285 (calcd. [M+H]^+^ ion for C_26_H_32_O_6_: *m*/*z* 441.2277; Δ 1.8 ppm). The NMR and mass spectra of compound **4** are available in the supplementary file (Figures S19–S23).
7-deacetyl-7-oxogedunin (**5**)—white powders, [α]^23^_D_ −62.0 (c 0.1; CHCl_3_). ^13^C-NMR (in CDCl_3_) see Table 2; ^1^H-NMR see Table 1. HRESITOF-MS: [M+H]^+^ ion *m*/*z* 439.2123 (calcd. [M+H]^+^ ion for C_26_H_30_O_6_: *m*/*z* 439.2121; Δ 0.5 ppm). The NMR and mass spectra of compound **5** are available in the supplementary file (Figures S24–S28).
12,20-dihydroxydammar-24-en-3-one (**6**)—white powders, [α]^23^_D_ +24.0 (c 0.1; CHCl_3_). ^13^C-NMR (in CDCl_3_) see Table 2; ^1^H-NMR see Table 1. HRESITOF-MS: [M+H]^+^ ion *m*/*z* 459.3835 (calcd. [M+H]^+^ ion for C_30_H_50_O_3_: *m*/*z* 459.3838; Δ 0.7 ppm). The NMR and mass spectra of compound **6** are available in the supplementary file (Figures S29–S33).

### 4.4. In Vitro Antibacterial Activity

The antibacterial activity of isolated compounds was evaluated using the disk diffusion method as a preliminary screening assay, as described in [36], against two Gram-positive (*E. faecalis* and *S. saprophyticus*) and two Gram-negative (*C. freundii* and *S. enterica*) bacteria.

### 4.5. DFT Calculation

Density Functional Theory (DFT) calculations were performed to achieve geometric optimization, and frequency analysis was conducted for isolated compounds (**1**–**7**) using the B3LYP/6-31+G(d,p) [37,38] level of theory with the Gaussian 16 software package [39]. The resulting data were visualized using GaussView 6.0.16 software [40]. The DFT results were used to analyze the quantum chemical characteristics, including the E_HOMO_, E_LUMO_, chemical hardness, softness, electronegativity, and chemical potential. Additionally, Koopman’s theorem [41] was applied to calculate the numerical values of several parameters, such as the ionization potential and electron affinity.

### 4.6. Molecular Docking

The crystal structure of Protein Tyrosine Phosphatase 1B (PTP1B) was obtained from the Protein Data Bank with PDB ID: 1NO6 (https://www.rcsb.org/structure/1no6, accessed on 4 April 2025) as the target protein [42]. The protein was protonated at pH 7.0 using the H++ web server (http://newbiophysics.cs.vt.edu/H++/, accessed on 4 April 2025). Meanwhile, 2-[(carboxycarbonyl)(1-naphthyl)amino]benzoic acid, a native ligand, was extracted, and its coordinates were used as a reference to identify the PTP1B pocket site. The modeled small molecules were prepared using the antechamber with the General Amber Force Field (GAFF) [43] to calculate the restrained charge, bonded, and nonbonded. Molecular docking was performed using the Dock6 package. The redocking step determined the pocket site according to the native ligand coordinates. Several crucial parameters were applied, including grid spacing (0.3 Å), center (X: 34.23, Y: 27.77, Z: 20.45), and dimensions (X: 28.57, Y: 28.08, and Z: 15.25 Å). It should be noted that an RMSD ≤ 2.0 Å is acceptable for redocking validation [44]. The validated parameters from the redocking step were applied to dock modeled small molecules to the PTP1B pocket site. The anchor-and-grow algorithm [45] was utilized for the ligand–protein energy interaction through the grid score (GS). In detail, the van der Waals (E_vdW_) and electrostatic (E_ele_) energies are described as the energy contributions in gas terms [44,45].

## 5. Conclusions

An ursane-type pentacyclic triterpene, (18β,19αH)-3-oxo-urs-12-en-27α-oic acid (**2**), has been isolated as a new natural compound from the tree bark of *S. koetjape*. Six known compounds, namely β-caryophyllene oxide (**1**), bryononic acid (**3**), 7-deacetylgedunin (**4**), 7-deacetyl-7-oxogedunin (**5**), 12,20-dihydroxydammar-24-en-3-one (**6**), and 12-hydroxydammarenolic acid (**7**), were also isolated from the MeOH extract of the tree bark. All compounds were assayed against *E. faecalis*, *S. saprophyticus*, *C. freundii,* and *S. enterica* bacteria. In addition, DFT analysis was performed to calculate the electronic properties of all the compounds. Of the compounds, only **7** was active against the four tested bacteria. The molecular interaction of **7** was examined using molecular docking, which revealed that the potent compound interacted well with the catalytic sites of the enzyme, which contributes to microbial growth.

## Figures and Tables

**Table 1:** ^13^C-NMR data of compounds **2**–**6** in CDCl_3_.

Position	2	3	4	5	6
1	39.3	35.4	157.8	155.9	39.8
2	34.0	34.4	125.8	126.4	34.1
3	217.9	218.2	205.6	203.2	217.8
4	47.1	47.1	44.2	45.2	47.4
5	54.6	51.2	44.6	54.6	55.3
6	19.7	20.6	27.3	36.7	19.7
7	36.0	34.2	69.7	208.2	34.1
8	39.8	134.9	43.7	53.4	51.6
9	45.9	132.7	38.0	47.6	49.4
10	36.6	30.9	40.2	39.6	36.8
11	22.9	20.5	15.1	17.2	31.5
12	128.4	30.0	26.4	32.2	70.7
13	133.2	37.1	38.4	37.7	48.0
14	55.8	42.2	70.1	65.6	39.7
15	22.4	25.2	57.8	53.6	31.0
16	28.9	27.7	168.3	166.9	26.5
17	33.7	37.5	78.5	78.0	53.4
18	60.3	44.5	17.8	20.9	15.4
19	37.7	29.5	19.9	19.8	15.9
20	39.6	40.4	120.6	120.2	74.7
21	30.4	30.5	141.2	141.0	27.1
22	40.8	36.8	110.0	109.8	34.3
23	27.0	21.1	143.0	143.1	22.4
24	21.3	26.8	-	-	124.8
25	16.4	19.5	-	-	132.0
26	18.1	21.7	-	-	25.8
27	180.9	18.1	-	-	17.8
28	29.0	31.1	27.3	27.0	21.0
29	17.8	185.1	21.5	20.6	26.7
30	21.4	32.7	18.7	17.4	16.8

**Table 2:** ^1^H-NMR data of compounds **2**–**6** in CDCl_3_.

Position	2	3	4	5	6
1	1.57 (*m*); 1.93 (*m*)	2.02 (*m*); 1.53 (*m*)	7.10 (*d*, 10.2)	7.11 (*d*, 10.2)	1.47 (*m*)
2	2.46 (*m*)	2.45 (*m*); 2.53(*m*)	5.84 (*d*, 10.2)	5.92 (*d*, 10.2)	2.44 (*m*); 2.51 (*m*)
5	1.35 (*m*)	1.61 (*m*)	2.48 (*m*)	2.18 (*m*)	1.36 (*m*)
6	1.43 (*m)*	1.95 (*m*)	1.66 (*m*); 1.90 (*m*)	2.41 (*dd*, 2.9; 13.9); 2.93 (*t*, 14.2)	1.52 (*m*)
7	1.16 (*m*); 1.75 (*m*)	2.03 (*m*); 0.89 (*m*)	3.57 (*s*)	-	1.34 (*m*); 1.55 (*m*)
9	2.33 (*dd*, 5.45; 11.0)	-	2.52 (*m*)	2.21 (*m*)	1.51 (*m*)
11	2.01 (*m*)	1.62 (*m*); 1.44 (*m*)	1.80 (*m*); 1.97 (*m*)	1.80 (*m*); 2.00 (*m*)	1.31 (*m*); 1.85 (*m*)
12	5.58 (*qui*, 2.4)	1.67 (*m*); 1.28 (*m*)	1.54 (*m*); 1.71 (*m*)	1.48 (*dt*, 12.43; 8.84); 1.86 (*m*)	3.60 (*td*, 10.41; 5.08)
13	-	-	-	-	1.76 (*t*, 10.65)
15	1.77 (*m*); 1.98 (*m*)	1.46 (*m*); 1.31 (*m*)	3.91 (*s*)	3.87 (*s*)	1.05 (*m*); 1.51 (*m*)
16	0.91 (*m*); 2.11 (*td*, 13.62; 3.85)	1.82 (*m*); 2.13 (*m*)	-	-	1.27 (*m*); 1.87 (*m*)
17	-	-	5.60 (*s*)	5.47 (*s*)	2.05 (*td*, 10.53; 7.16)
18	1.37 (*m*)	1.51 (*m*)	1.24 (*s*)	1.13 (*s*)	1.03 (*s*)
19	0.89 (*m*)	2.18 (*m*); 1.38 (*m*)	1.20 (*s*)	1.36 (*s*)	0.98 (*s*)
20	0.86 (*m*)	-	-	-	-
21	1.24 (*m*); 1.34 (*m*)	2.43 (*m*); 1.64 (*m*)	7.41 (*s*)	7.41 (*s*)	1.21 (*s*)
22	1.24 (*m*); 1.41 (*m*)	1.31 (*m*); 1.70 (*m*)	6.35 (*s*)	6.36 (*s*)	1.41 (*m*); 1.68 (*m*)
23	1.06 (*s*)	1.06 (*s*)	7.40 (*d*, 1.70)	7.39 (*d*, 0.95)	2.05 (*m*); 2.17 (*m*)
24	1.03 (*s*)	1.09 (*s*)	-	-	5.17 (*t*, 7.04)
25	1.05 (*s*)	1.03 (*s*)	-	-	-
26	1.07 (*s*)	0.96 (*s*)	-	-	1.70 (*s*)
27	-	0.85 (*s*)	-	-	1.64 (*s*)
28	0.84 (*s*)	1.03 (*s*)	1.14 (*s*)	1.16 (*s*)	1.05 (*s*)
29	0.79 (*d*, 5.5)	-	1.10 (*s*)	1.14 (*s*)	1.08 (*s*)
30	0.86 (*brs*)	1.22 (*s*)	1.09 (*s*)	1.22 (*s*)	0.89 (*s*)

**Table 3:** Antibacterial activities of Compounds **1**–**7**.

Compounds	Inhibition Zone Diameter (mm)			
	Gram-Positive Bacteria		Gram-Negative Bacteria	
	*E. faecalis*	*S. saprophyticus*	*S. enterica*	*C. freundii*
Amoxicillin ^(1)^	28	14	15	30
Chloramphenicol ^(1)^	8	25	26	25
**1**	-	-	-	-
**2**	-	-	-	-
**3**	-	-	-	-
**4**	-	-	-	-
**5**	-	-	-	-
**6**	-	-	-	-
**7**	9	7.5	8	9
5% DMSO-MHB ^(2)^	-	-	-	-

^(1)^ Positive control. ^(2)^ Solvent, negative control.

**Table 4:** Calculated molecular orbital energies and chemical reactivity descriptors.

Compound	E_HOMO_	E_LUMO_	IP	EA	χ	µ	η	ω	E_g_
**1**	−6.487	−0.142	6.49	0.14	3.31	−3.31	3.17	1.73	6.34
**2**	−6.565	−0.736	6.56	0.74	3.65	−3.65	2.91	2.29	5.83
**3**	−6.174	−0.587	6.17	0.59	3.38	−3.38	2.79	2.05	5.59
**4**	−6.667	−1.967	6.67	1.97	4.32	−4.32	2.35	3.96	4.70
**5**	−6.740	−2.195	6.74	2.20	4.47	−4.47	2.27	4.39	4.54
**6**	−6.351	−0.756	6.35	0.76	3.55	−3.55	2.80	2.26	5.59
**7**	−6.331	−0.458	6.33	0.46	3.39	−3.39	2.94	1.96	5.87

Ionization potential (IP), electron affinity (EA), electronegativity (χ), chemical potential (µ), chemical hardness (η), electrophilicity (ω), HOMO-LUMO energy gap (E_g_). All values are in eV units.

## Data Availability

The original contributions presented in this study are included in the article/Supplementary Materials. Further inquiries can be directed to the corresponding authors.

## Supplementary Materials

The following supporting information can be downloaded at https://www.mdpi.com/article/10.3390/ijms262110389/s1.
