# Supplementary material for: A New Ursane-Type Pentacyclic Triterpenoid from the Tree Bark of Sandoricum koetjape: Antibacterial, DFT, and Molecular Docking Study
Source: Int J Mol Sci. 2025 Oct 25;26(21):10389. doi: 10.3390/ijms262110389 (PMC12607651; doi:10.3390/ijms262110389)
Supplement: Supplementary file 1 [file ijms-26-10389-s001.zip › ijms-3924630-supplementary.pdf]

Supplementary Materials

Figure S1 ^{1}H NMR ($CDCl_3$, 500 MHz) spectrum of **1** 2
Figure S2 ^{13}C NMR ($CDCl_3$, 125 MHz) spectrum of **1** 2
Figure S3 HSQC ($CDCl_3$, 500 MHz, 125 MHz) spectrum of **1** 3
Figure S4 HMBC ($CDCl_3$, 500 MHz, 125 MHz) spectrum of **1** 3
Figure S5 HRESIMS spectrum of **1** 4
Figure S6 ^{1}H NMR ($CDCl_3$, 500 MHz) spectrum of **2** 4
Figure S7 ^{1}H NMR ($CDCl_3$, 500 MHz) spectrum of **2** (from 0.5 to 2.6 ppm) 5
Figure S8 ^{13}C NMR ($CDCl_3$, 125 MHz) spectrum of **2** 5
Figure S9 HSQC ($CDCl_3$, 500 MHz, 125 MHz) spectrum of **2** 6
Figure S10 HMBC ($CDCl_3$, 500 MHz, 125 MHz) spectrum of **2** 6
Figure S11 ROESY ($CDCl_3$, 500 MHz) spectrum of **2** 7
Figure S12 HRESIMS spectrum of **2** 8
Figure S13 ^{1}H NMR ($CDCl_3$, 500 MHz) spectrum of **3** 8
Figure S14 ^{1}H NMR ($CDCl_3$, 500 MHz) spectrum of **3** (from 0.5 to 2.6 ppm) 9
Figure S15 ^{13}C NMR ($CDCl_3$, 125 MHz) spectrum of **3** 9
Figure S16 HSQC ($CDCl_3$, 500 MHz, 125 MHz) spectrum of **3** 10
Figure S17 HMBC ($CDCl_3$, 500 MHz, 125 MHz) spectrum of **3** 11
Figure S18 HRESIMS spectrum of **3** 11
Figure S19 ^{1}H NMR ($CDCl_3$, 500 MHz) spectrum of **4** 12
Figure S20 ^{13}C NMR ($CDCl_3$, 125 MHz) spectrum of **4** 12
Figure S21 HSQC ($CDCl_3$, 500 MHz, 125 MHz) spectrum of **4** 13
Figure S22 HMBC ($CDCl_3$, 500 MHz, 125 MHz) spectrum of **4** 13
Figure S23 HRESIMS spectrum of **4** 14
Figure S24 ^{1}H NMR ($CDCl_3$, 500 MHz) spectrum of **5** 14
Figure S25 ^{13}C NMR ($CDCl_3$, 125 MHz) spectrum of **5** 15
Figure S26 HSQC ($CDCl_3$, 500 MHz, 125 MHz) spectrum of **5** 15
Figure S27 HMBC ($CDCl_3$, 500 MHz, 125 MHz) spectrum of **5** 16
Figure S28 HRESIMS spectrum of **5** 16
Figure S29 ^{1}H NMR ($CDCl_3$, 500 MHz) spectrum of **6** 17
Figure S30 ^{13}C NMR ($CDCl_3$, 125 MHz) spectrum of **6** 17
Figure S31 HSQC ($CDCl_3$, 500 MHz, 125 MHz) spectrum of **6** 18
Figure S32 HMBC ($CDCl_3$, 500 MHz, 125 MHz) spectrum of **6** 18
Figure S33 HRESIMS spectrum of **6** 19

Table S1 Key NOESY or ROESY correlation of **2** 7

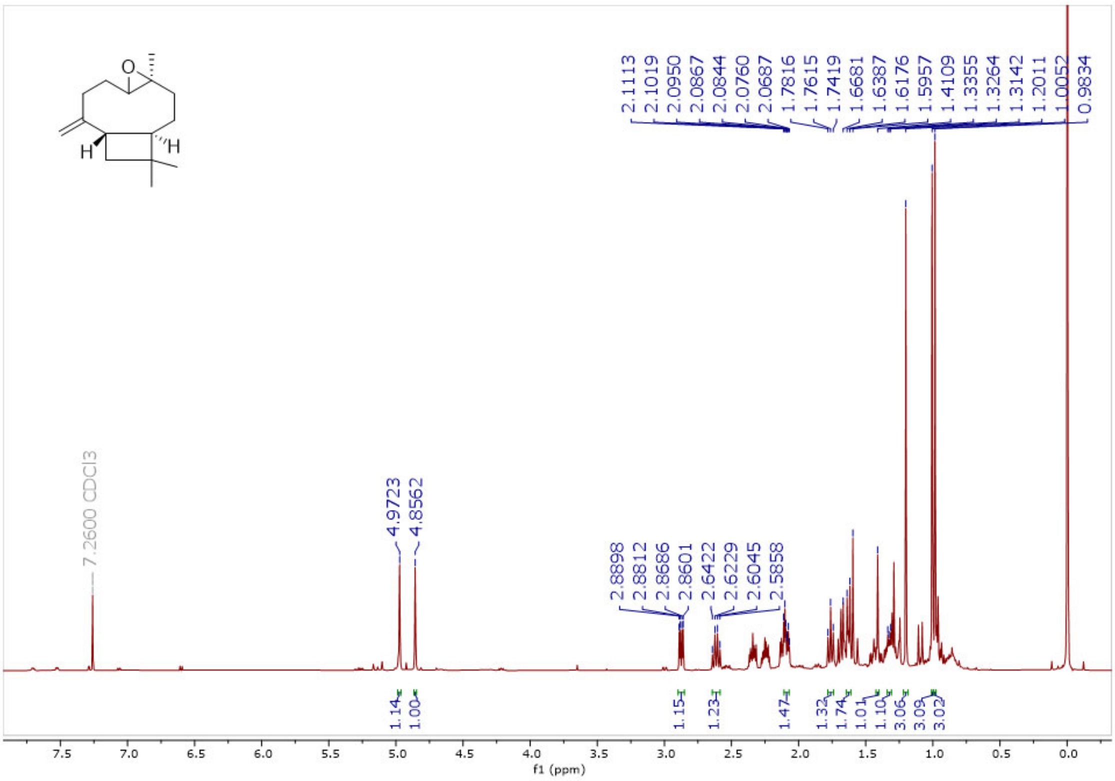

Figure S1 ^{1}H NMR ($CDCl_3$, 500 MHz) spectrum of **1**

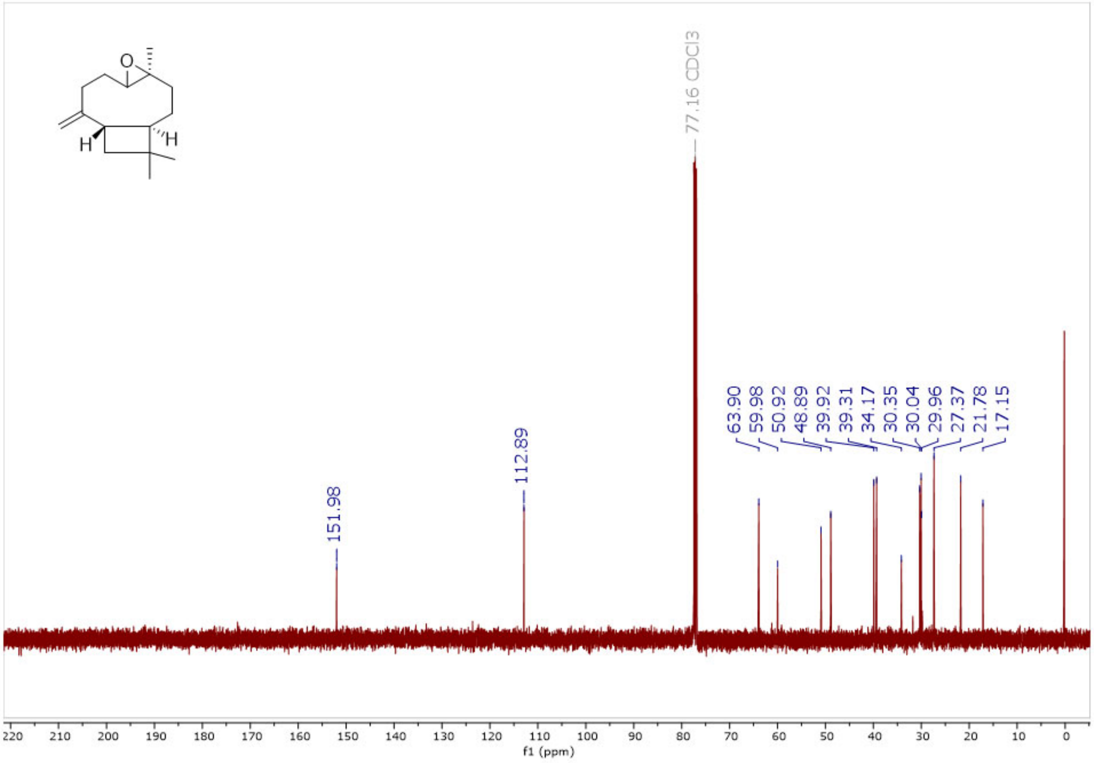

Figure S2 ^{13}C NMR ($CDCl_3$, 125 MHz) spectrum of **1**

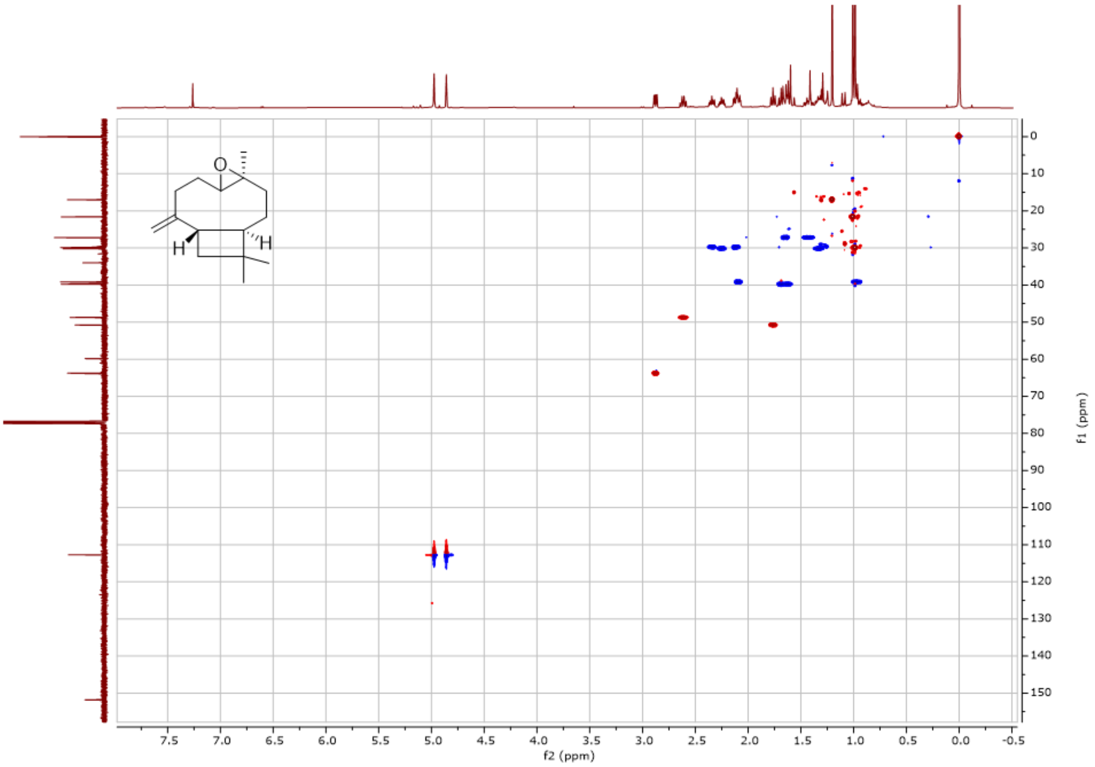

Figure S3 HSQC ($CDCl_3$, 500 MHz, 125 MHz) spectrum of **1**

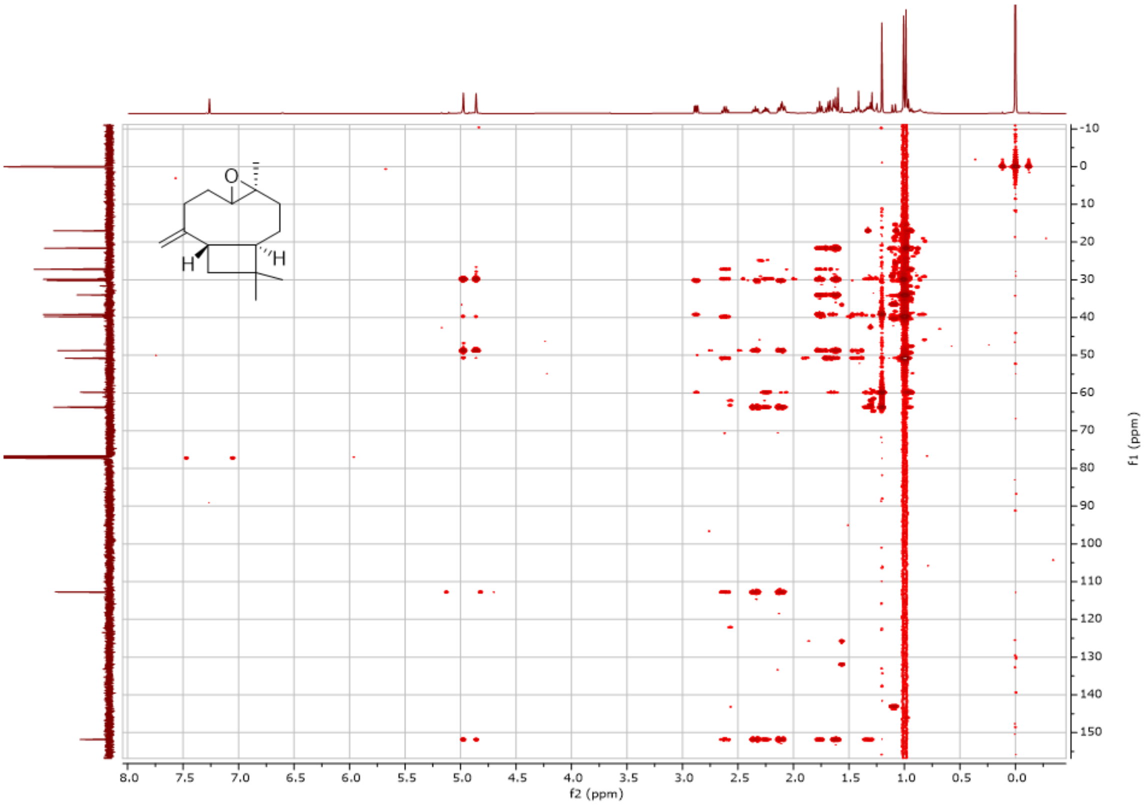

Figure S4 HMBC ($CDCl_3$, 500 MHz, 125 MHz) spectrum of **1**

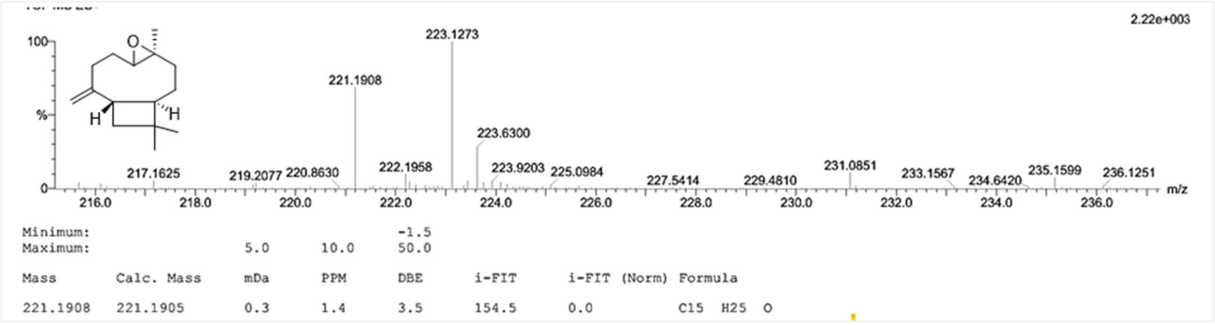

Figure S5 HRESIMS spectrum of **1**

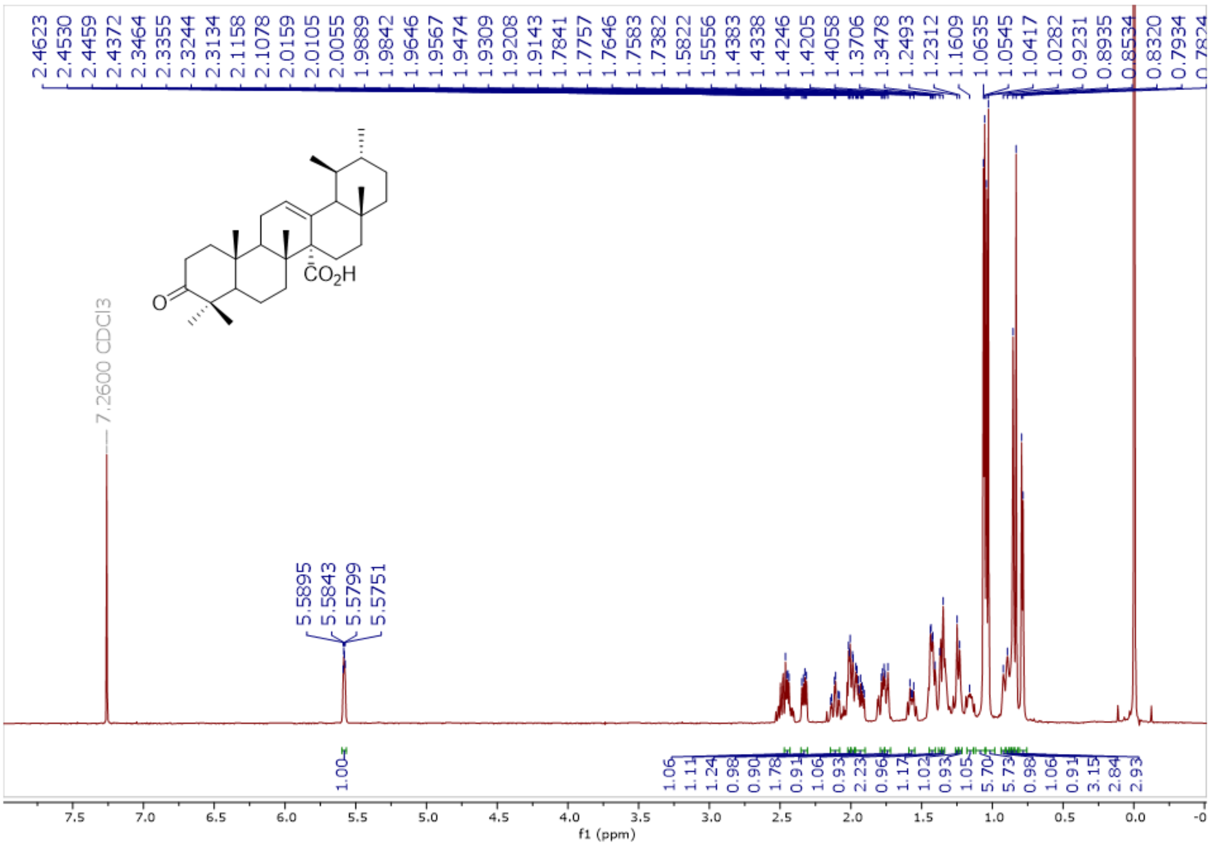

Figure S6 ^{1}H NMR ($CDCl_3$, 500 MHz) spectrum of **2**

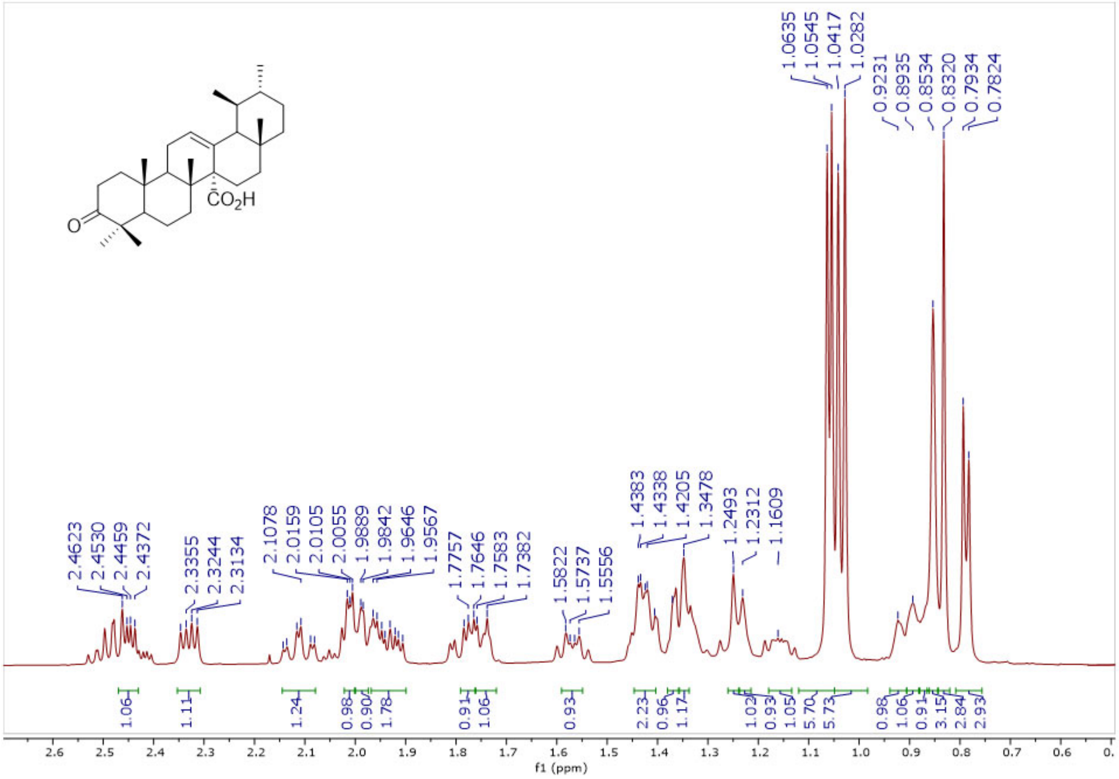

Figure S7 ^{1}H NMR ($CDCl_3$, 500 MHz) spectrum of **2** (from 0.5 to 2.6 ppm)

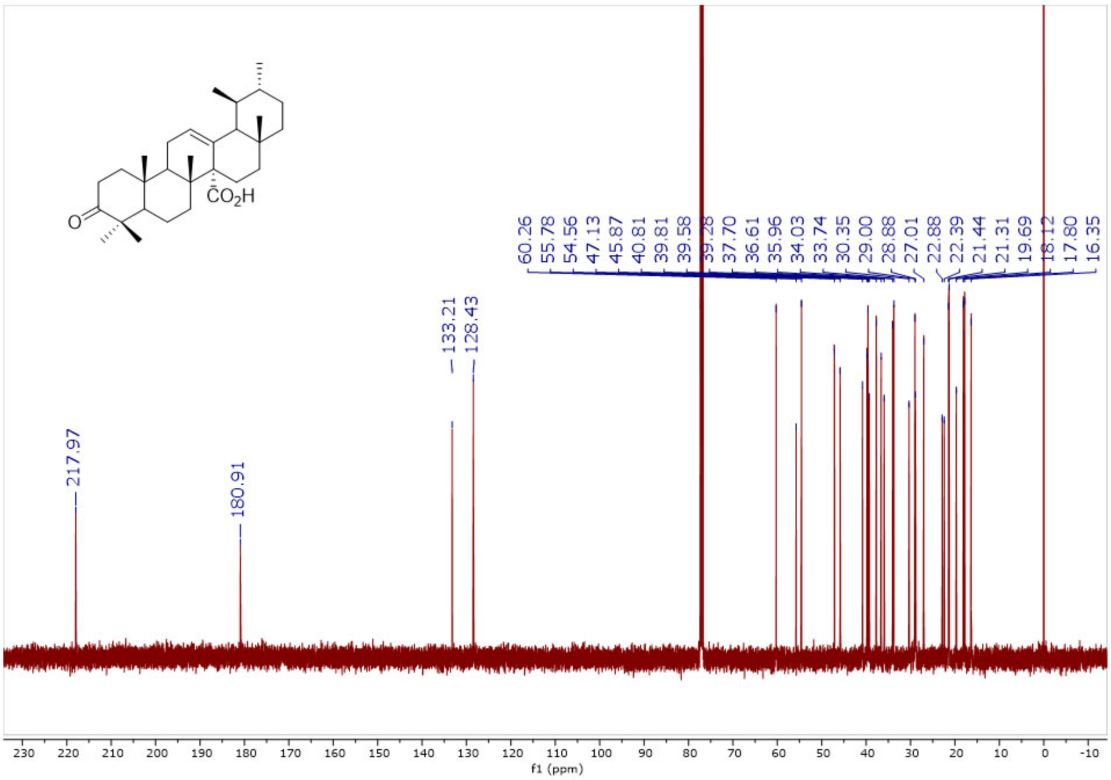

Figure S8 ^{13}C NMR ($CDCl_3$, 125 MHz) spectrum of **2**

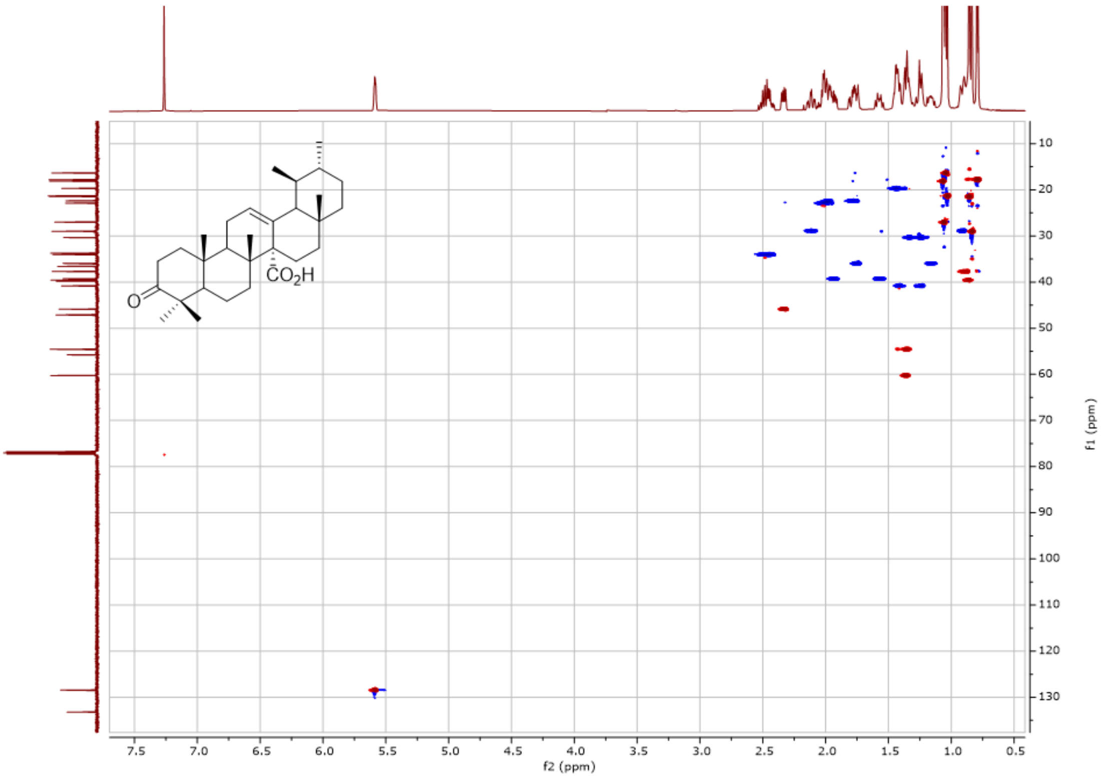

Figure S9 HSQC (CDCl3, 500 MHz, 125 MHz) spectrum of **2**

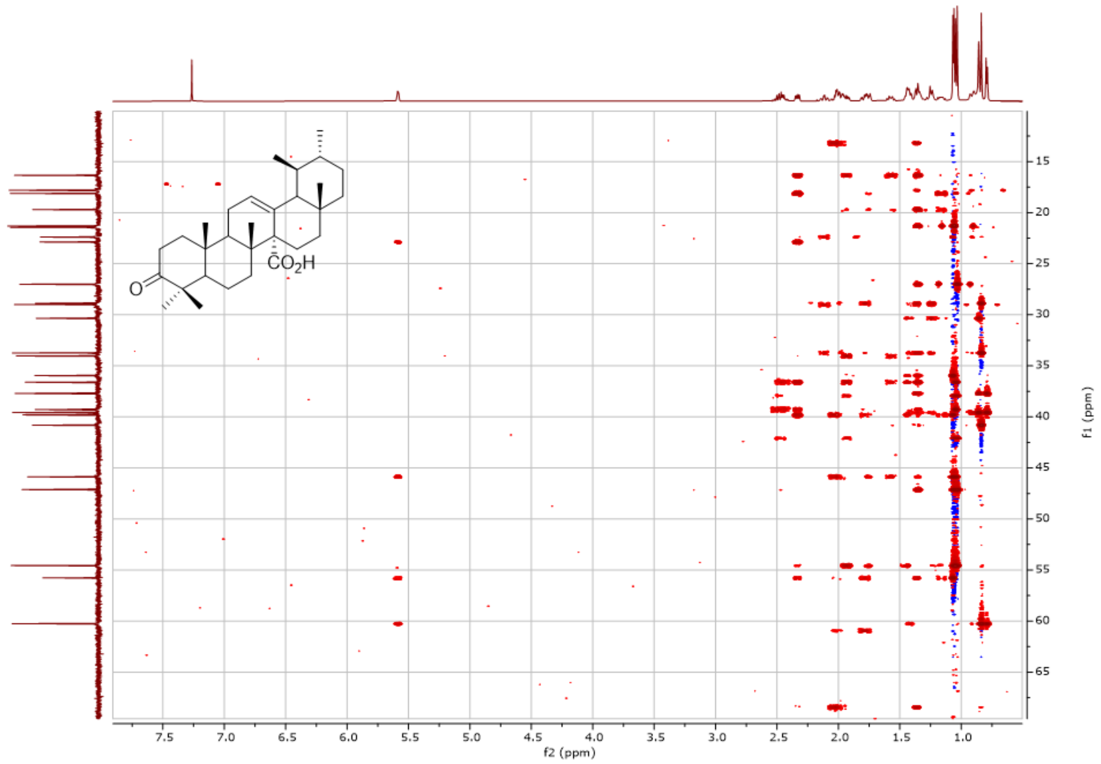

Figure S10 HMBC (CDCl3, 500 MHz, 125 MHz) spectrum of **2**

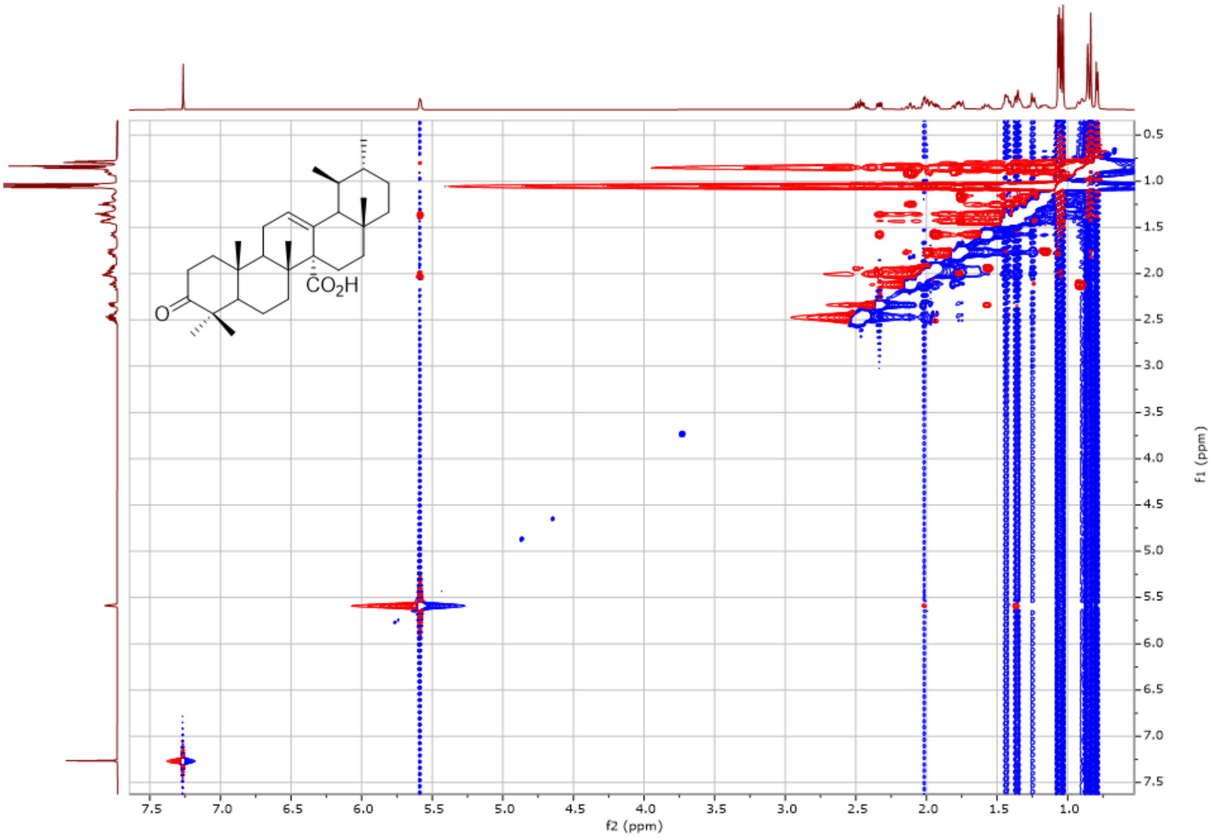

Figure S11 ROESY ($CDCl_3$, 500 MHz) spectrum of **2**

Table S1 Key NOESY or ROESY correlation of **2**

^{1}H signal (δ_H)	Assignment	H correlation with
5.58	H-12	0.79 (H-29)<br>2.01 (H-11β)
2.33	H-9	1.35 (H-5)<br>1.57 (H-1α)<br>2.01 (H-11α)
2.01	H-11β	1.05 (H-25)
1.77	H-15β	0.84 (H-28)<br>1.16 (H-7β)<br>1.07 (H-26)
1.75	H-7α	1.43 (H-6α)
1.35	H-5	1.06 (H-23)
1.37	H-18	0.84 (H-28)<br>0.86 (H-20)
2.11	H-16α	0.89 (H-19)

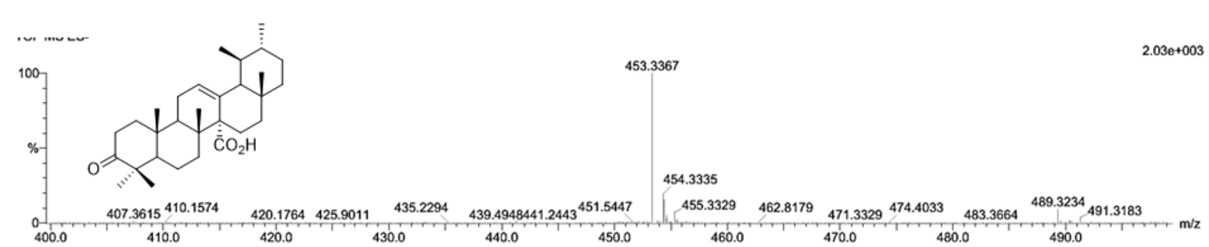

Minimum:				-1.5			
Maximum:		5.0	10.0	50.0			
Mass	Calc. Mass	mDa	PPM	DBE	i-FIT	i-FIT (Norm)	Formula
453.3367	453.3369	-0.2	-0.4	8.5	116.0	0.0	C30 H45 O3

Figure S12 HRESIMS spectrum of **2**

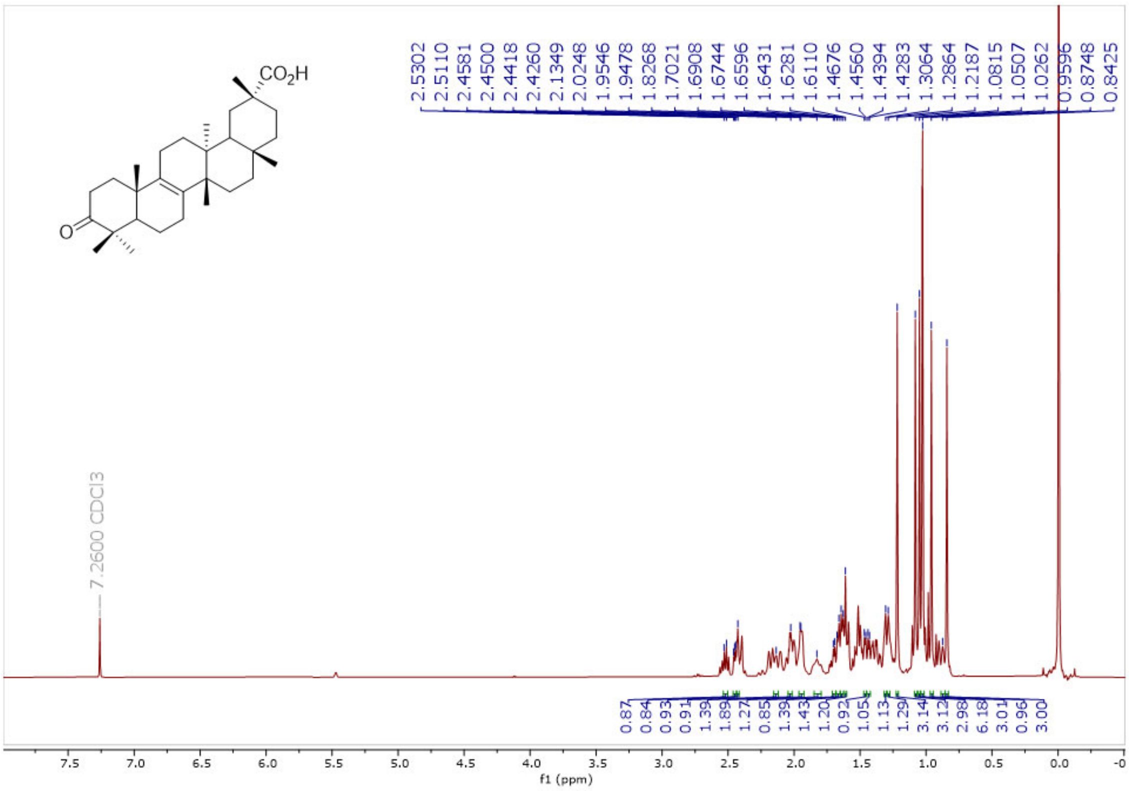

Figure S13 ^{1}H NMR ($CDCl_3$, 500 MHz) spectrum of **3**

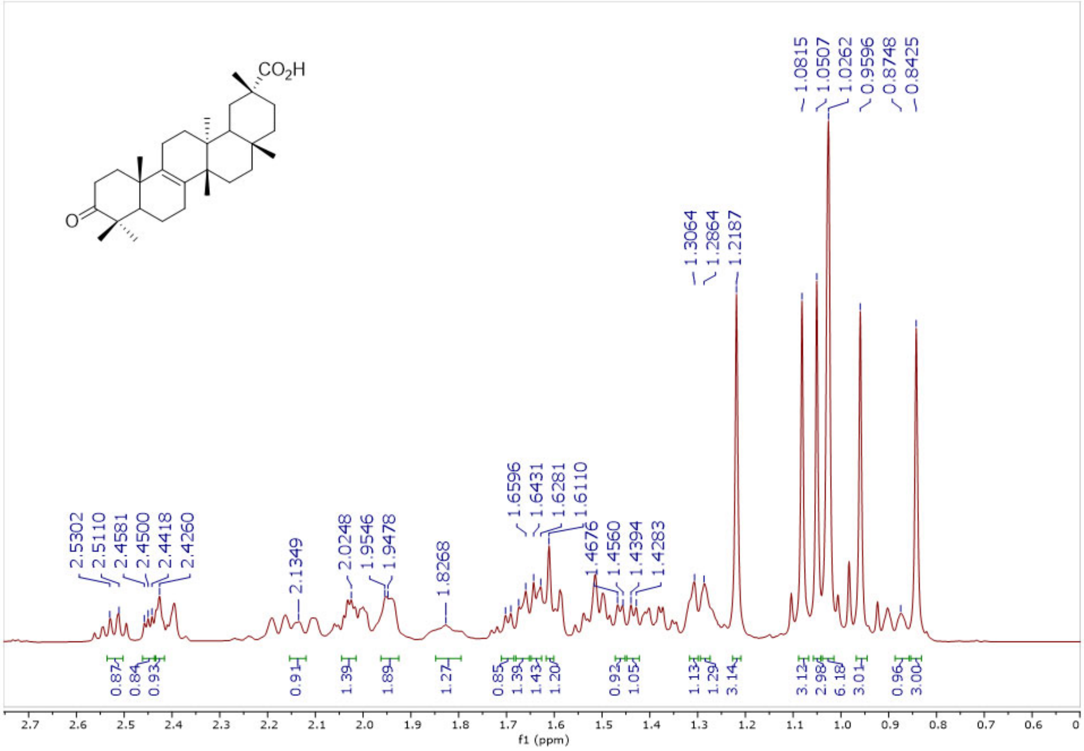

Figure S14 ^{1}H NMR ($CDCl_3$, 500 MHz) spectrum of **3** (from 0.5 to 2.6 ppm)

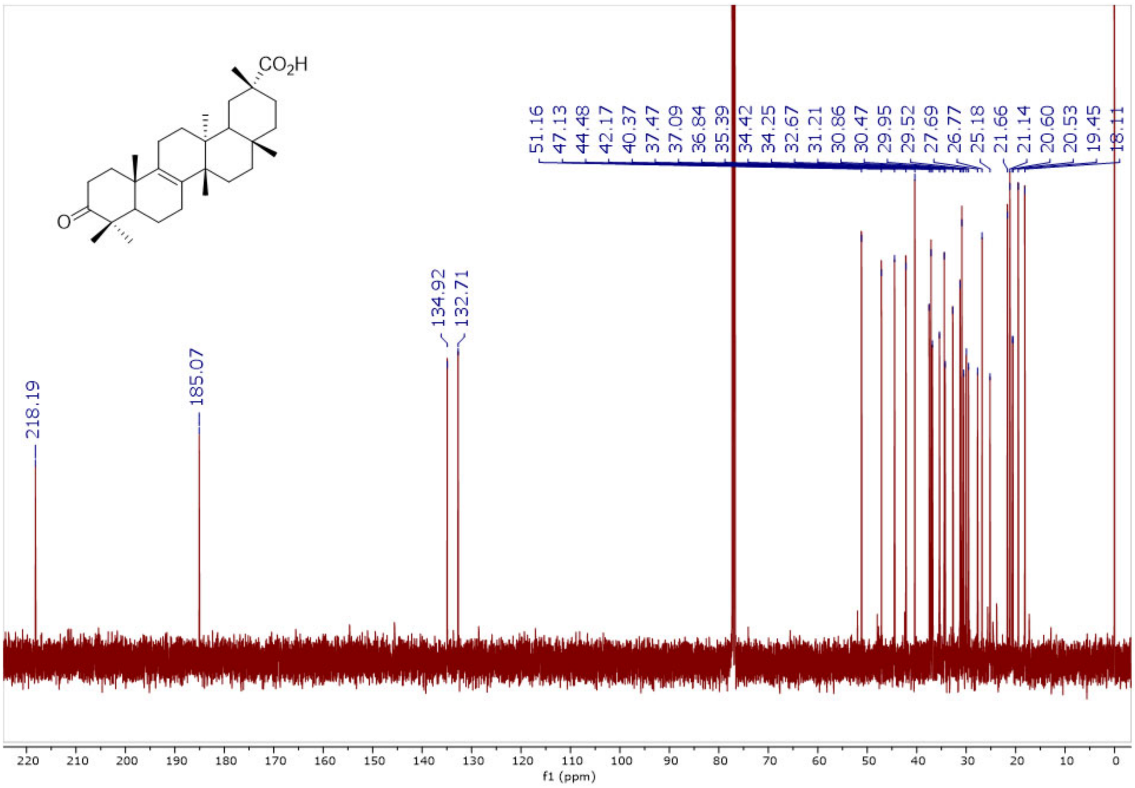

Figure S15 ^{13}C NMR ($CDCl_3$, 125 MHz) spectrum of **3**

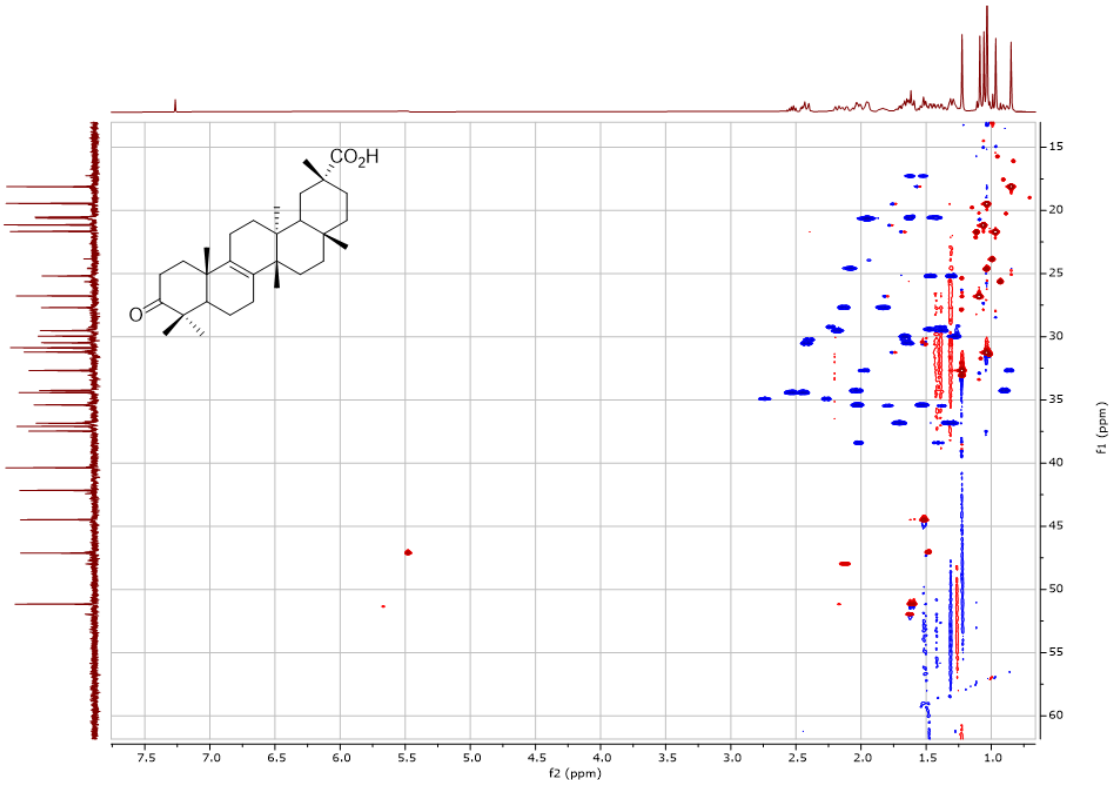

Figure S16 HSQC ($CDCl_3$, 500 MHz, 125 MHz) spectrum of **3**

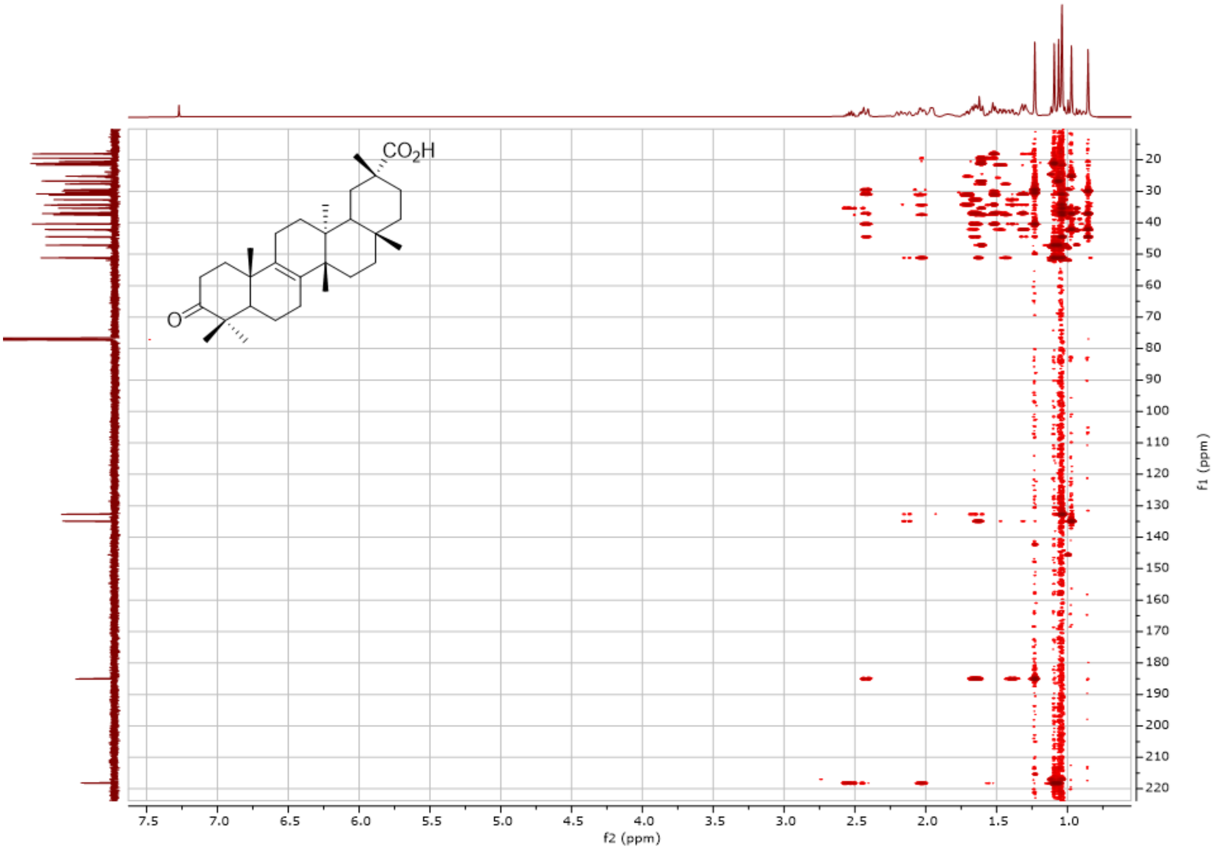

Figure S17 HMBC ($CDCl_3$, 500 MHz, 125 MHz) spectrum of **3**

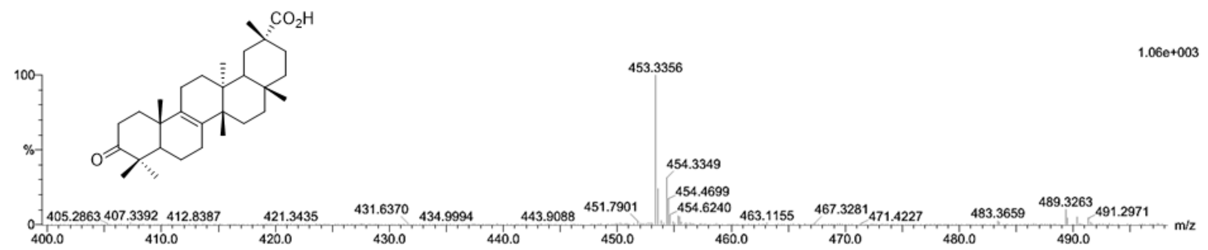

Minimum:				-1.5			
Maximum:		5.0	10.0	50.0			
Mass	Calc. Mass	mDa	PPM	DBE	i-FIT	i-FIT (Norm)	Formula
453.3356	453.3369	-1.3	-2.9	8.5	79.0	0.0	C30 H45 O3

Figure S18 HRESIMS spectrum of **3**

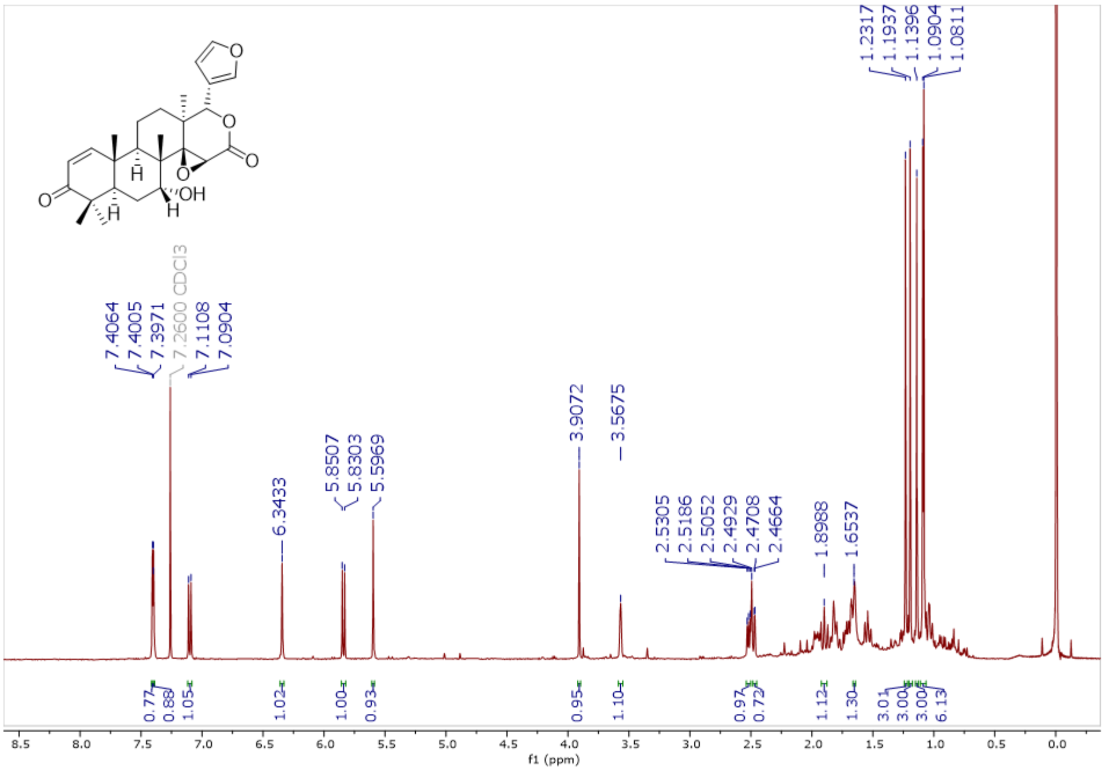

Figure S19 ^{1}H NMR ($CDCl_3$, 500 MHz) spectrum of **4**

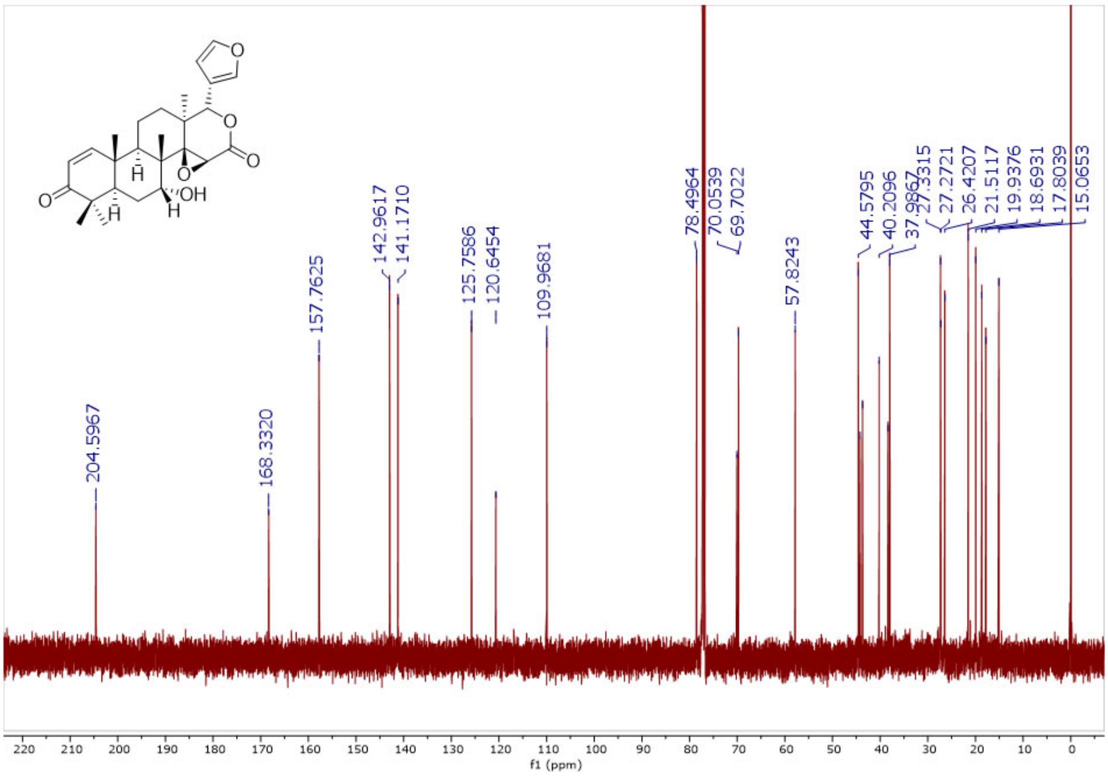

Figure S20 ^{13}C NMR ($CDCl_3$, 125 MHz) spectrum of **4**

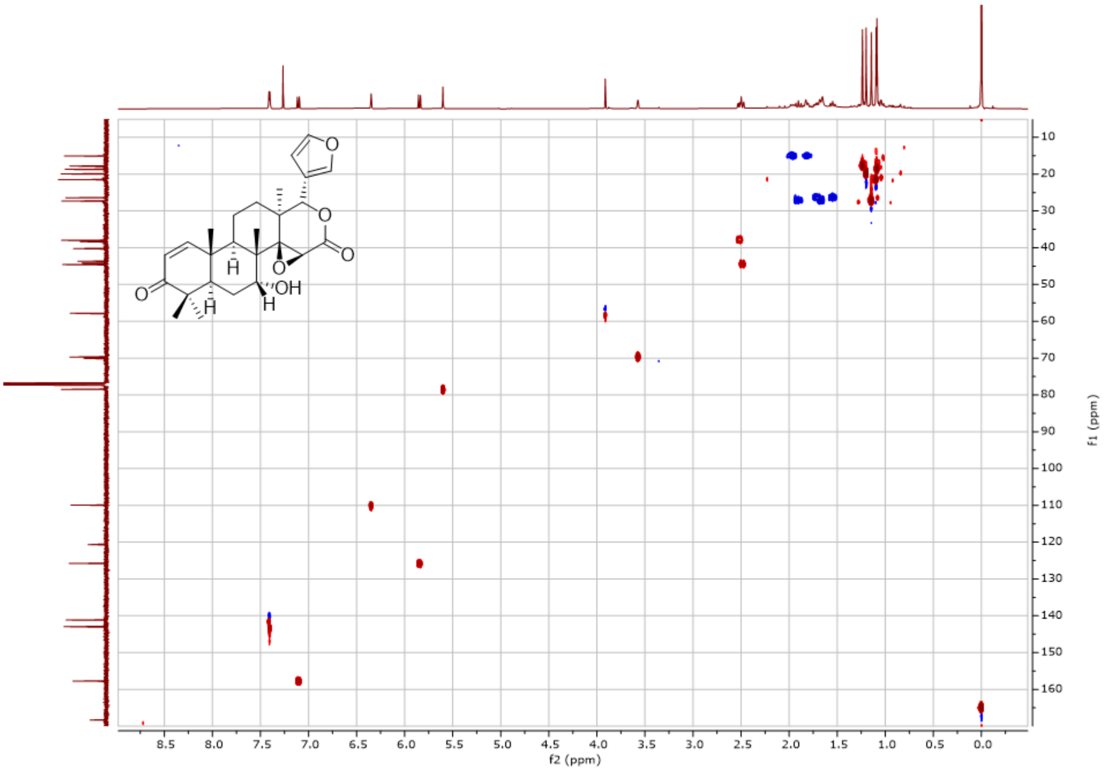

Figure S21 HSQC ($CDCl_3$, 500 MHz, 125 MHz) spectrum of **4**

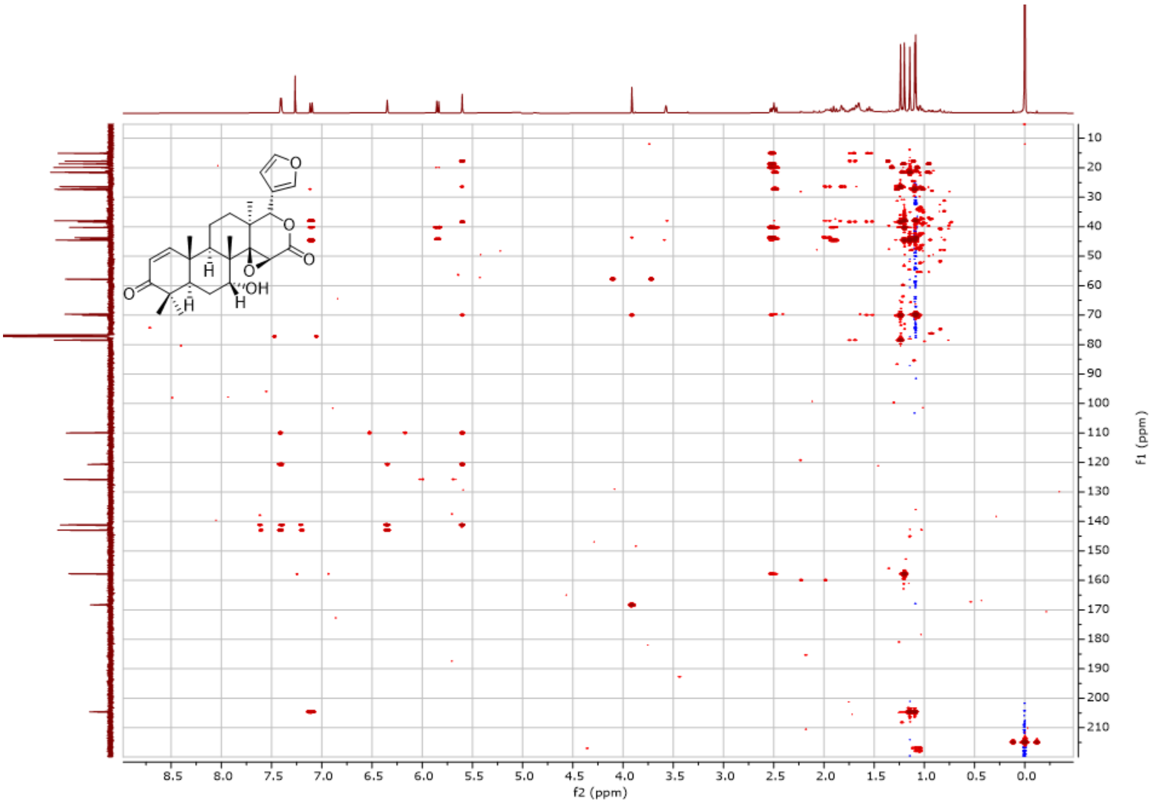

Figure S22 HMBC ($CDCl_3$, 500 MHz, 125 MHz) spectrum of **4**

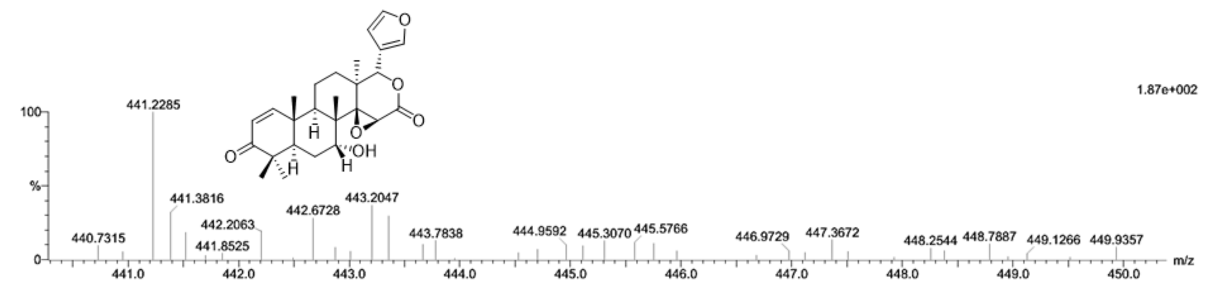

Minimum:				-1.5			
Maximum:		5.0	10.0	50.0			
Mass	Calc. Mass	mDa	PPM	DBE	i-FIT	i-FIT (Norm)	Formula
441.2285	441.2277	0.8	1.8	10.5	78.0	0.0	C26 H33 O6

Figure S23 HRESIMS spectrum of **4**

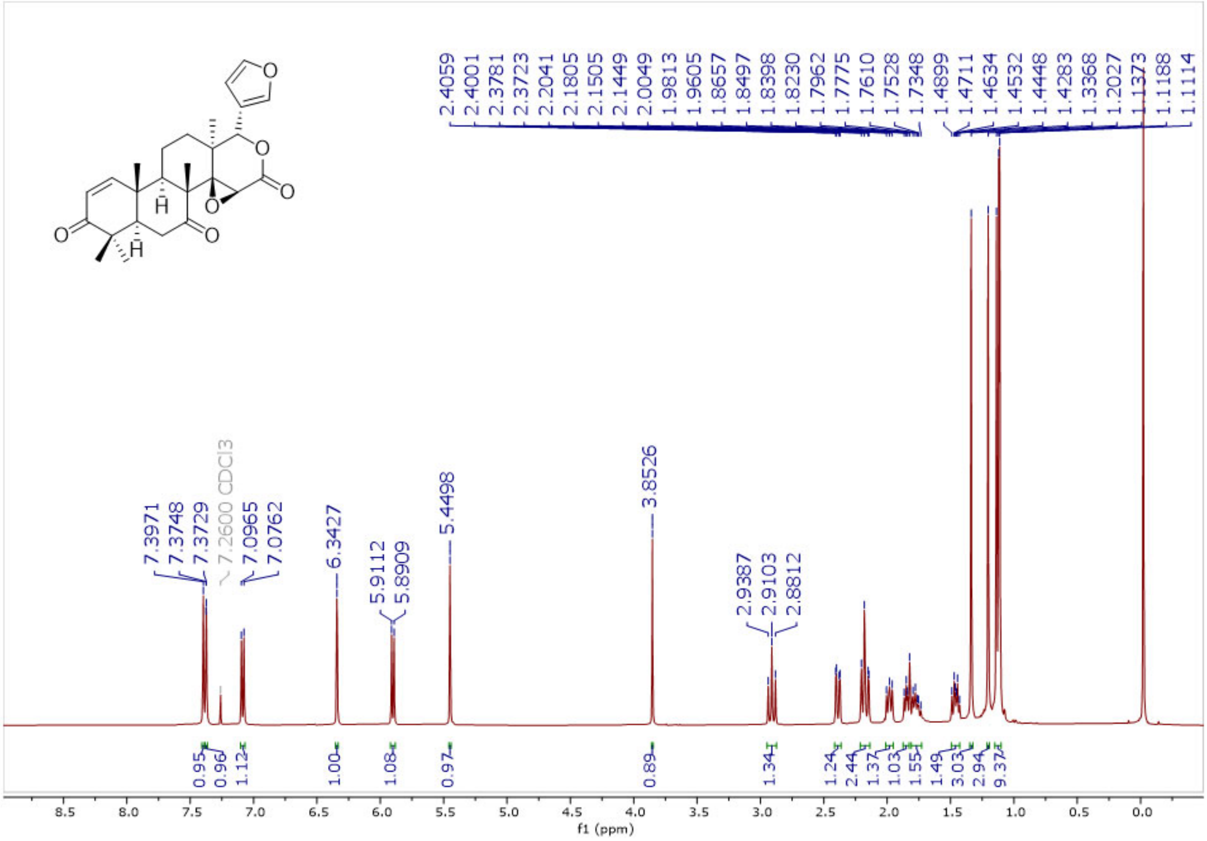

Figure S24 ^{1}H NMR ($CDCl_3$, 500 MHz) spectrum of **5**

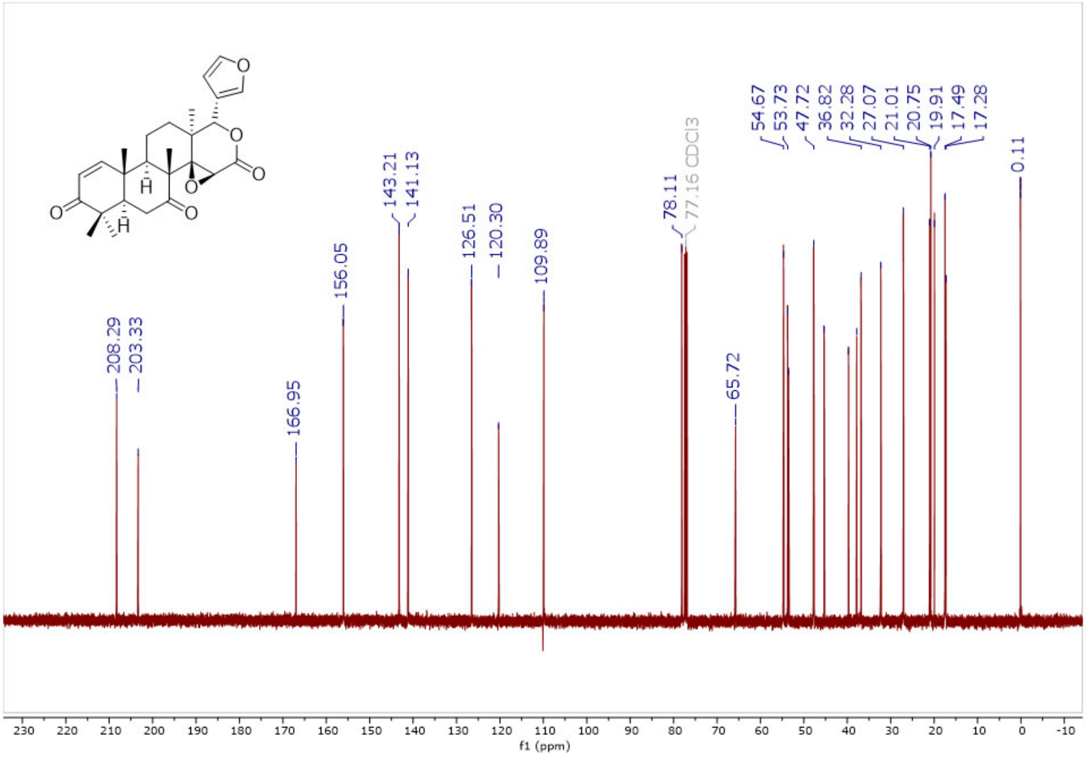

Figure S25 ^{13}C NMR ($CDCl_3$, 125 MHz) spectrum of **5**

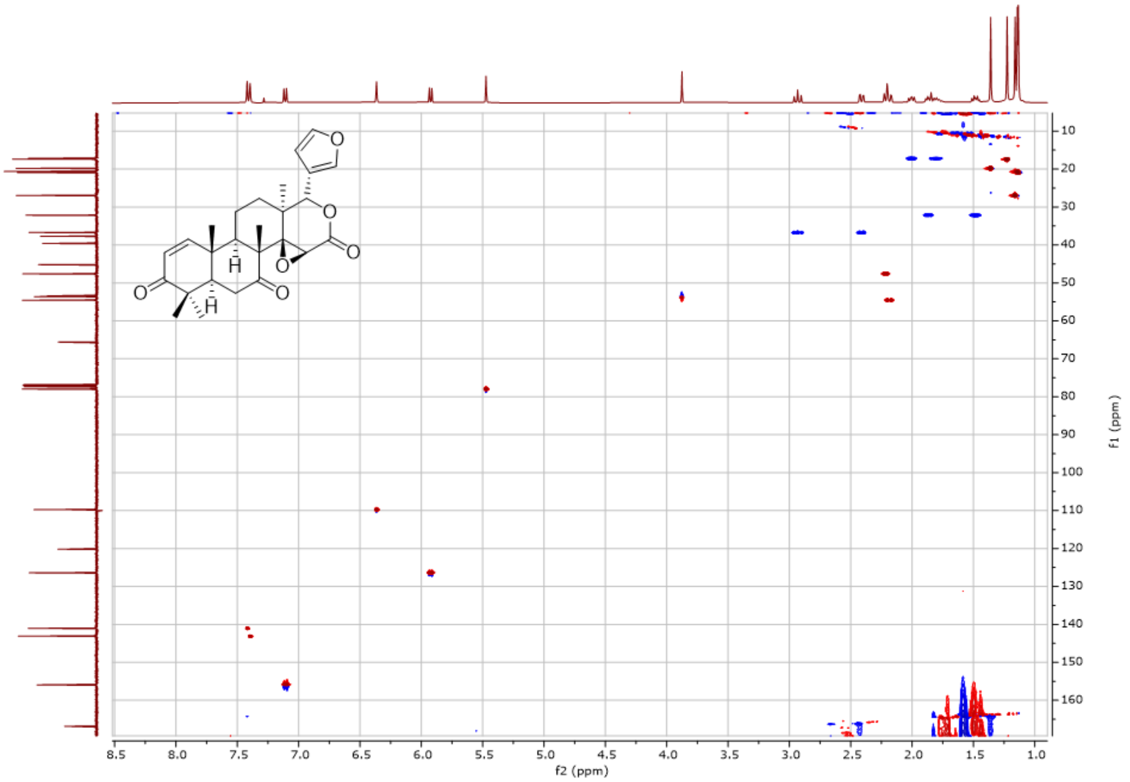

Figure S26 HSQC ($CDCl_3$, 500 MHz, 125 MHz) spectrum of **5**

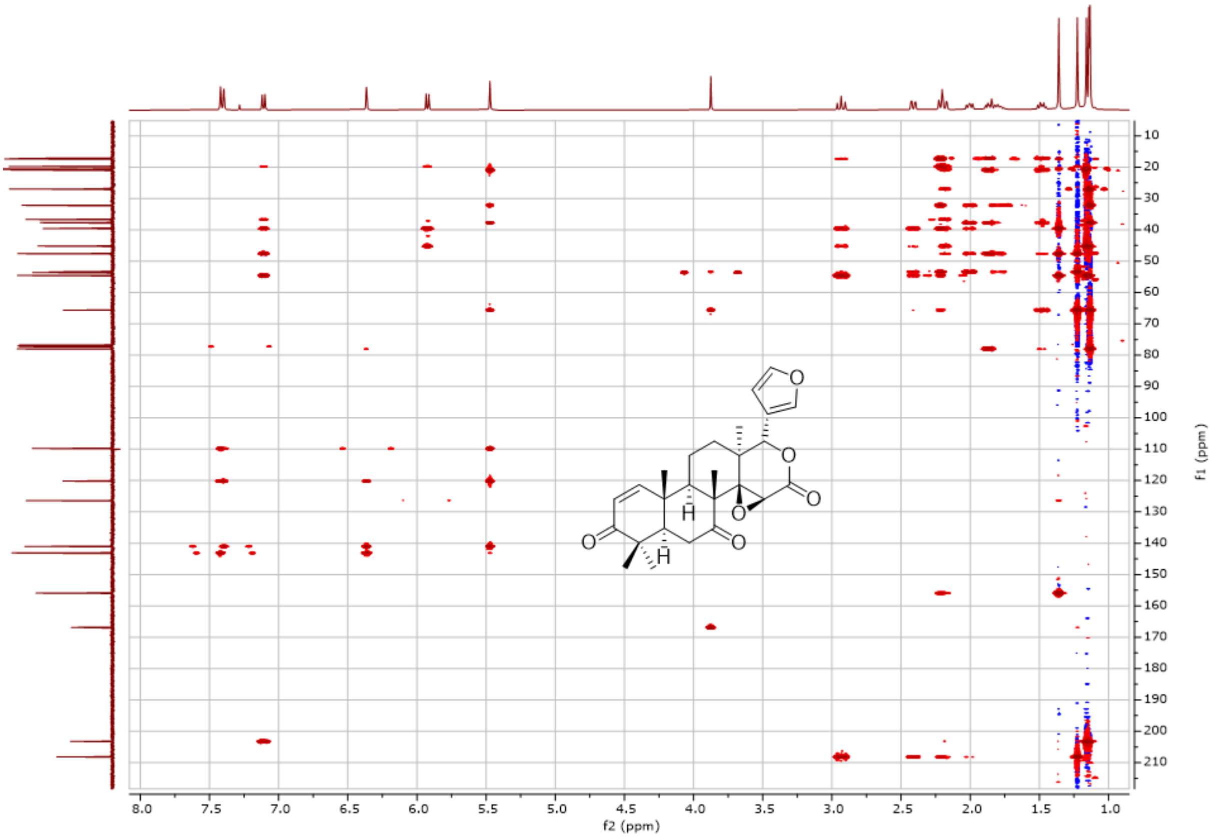

Figure S27 HMBC ($CDCl_3$, 500 MHz, 125 MHz) spectrum of **5**

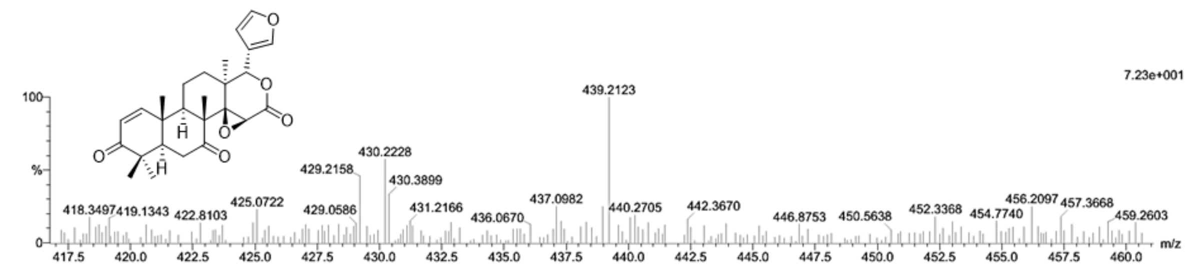

Minimum:				-1.5			
Maximum:		5.0	10.0	50.0			
Mass	Calc. Mass	mDa	PPM	DBE	i-FIT	i-FIT (Norm)	Formula
439.2123	439.2121	0.2	0.5	11.5	54.2	0.0	C26 H31 O6

Figure S28 HRESIMS spectrum of **5**

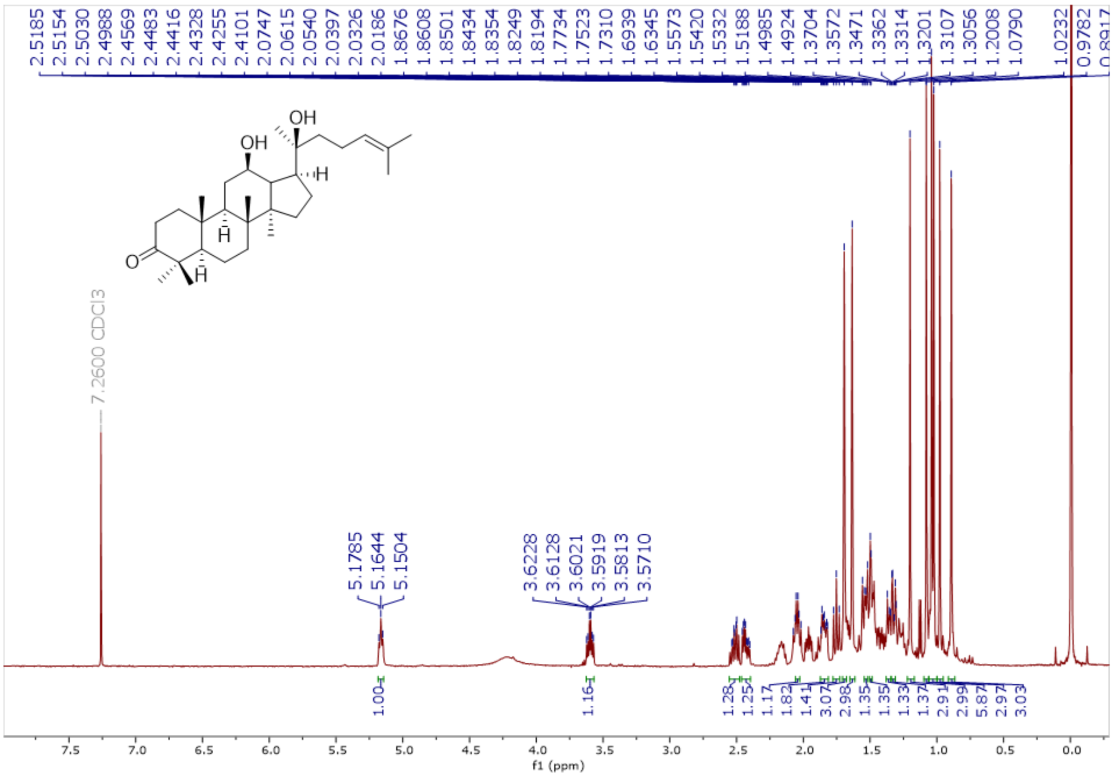

Figure S29 ^{1}H NMR ($CDCl_3$, 500 MHz) spectrum of **6**

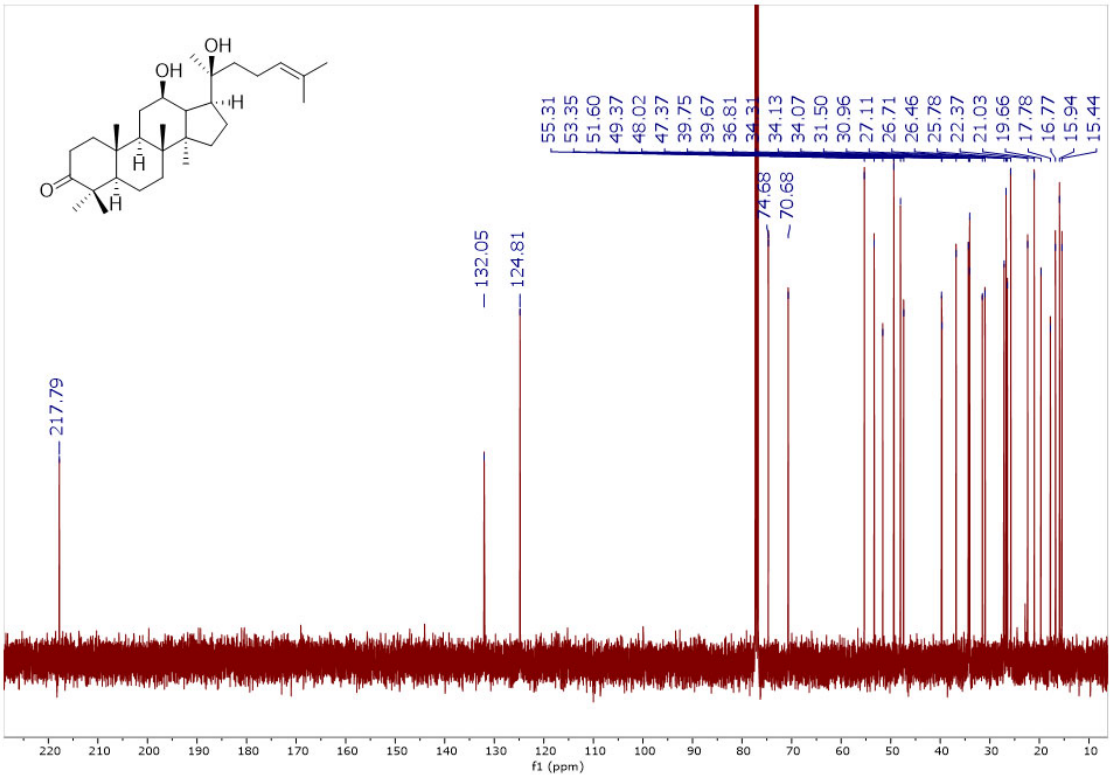

Figure S30 ^{13}C NMR ($CDCl_3$, 125 MHz) spectrum of **6**

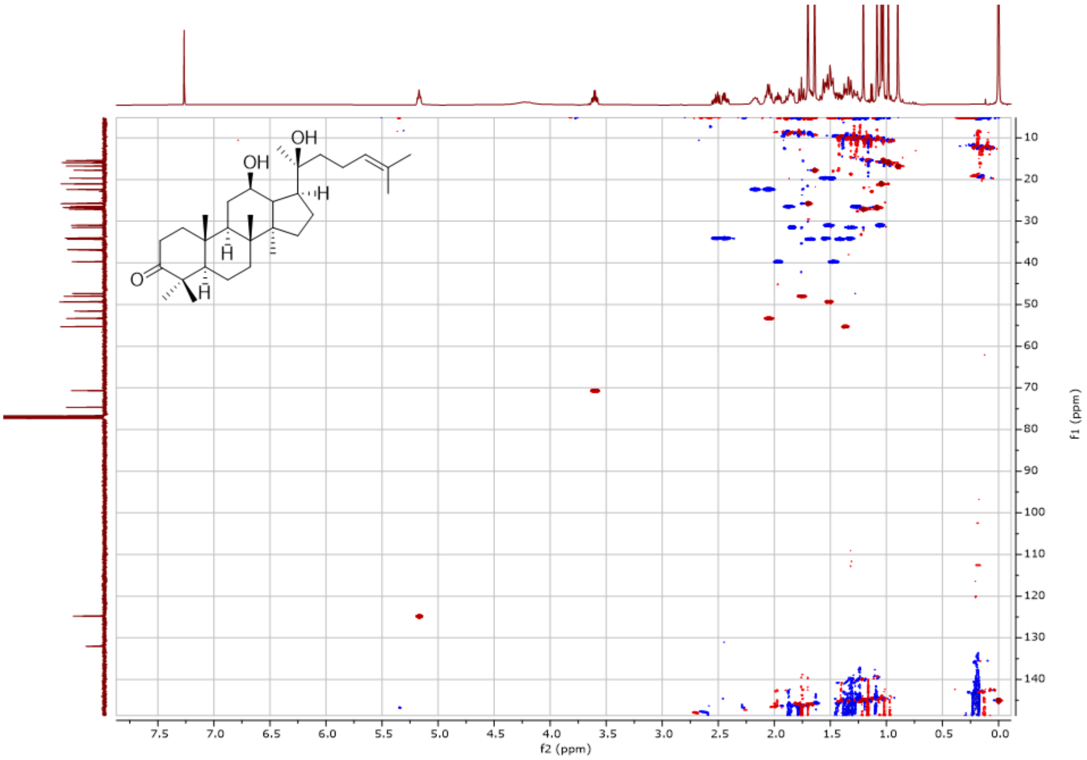

Figure S31 HSQC ($CDCl_3$, 500 MHz, 125 MHz) spectrum of **6**

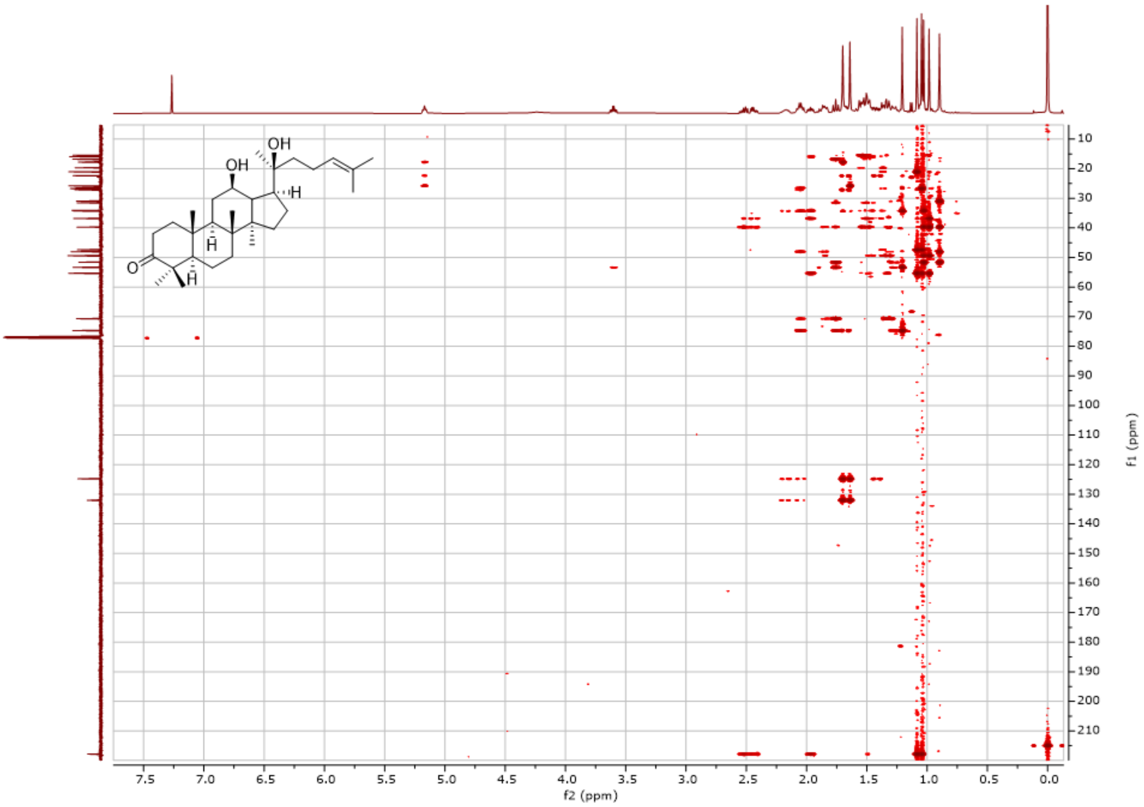

Figure S32 HMBC ($CDCl_3$, 500 MHz, 125 MHz) spectrum of **6**

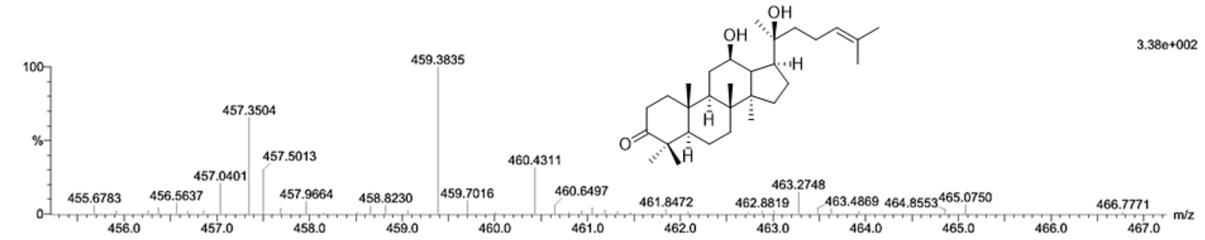

Minimum:				-1.5			
Maximum:		5.0	10.0	50.0			
Mass	Calc. Mass	mDa	PPM	DBE	i-FIT	i-FIT (Norm)	Formula
459.3835	459.3838	-0.3	-0.7	5.5	63.9	0.0	C30 H51 O3

Figure S33 HRESIMS spectrum of **6**
